# Antipsychotic Medication Use Among Older Adults Following Infection-Related Hospitalization

**DOI:** 10.1001/jamanetworkopen.2023.0063

**Published:** 2023-02-17

**Authors:** Yichi Zhang, James M. Wilkins, Lily Gui Bessette, Cassandra York, Vincent Wong, Kueiyu Joshua Lin

**Affiliations:** 1 Department of Epidemiology, Harvard T. H. Chan School of Public Health, Boston, Massachusetts; 2 Division of Pharmacoepidemiology and Pharmacoeconomics, Department of Medicine, Brigham and Women’s Hospital, Harvard Medical School, Boston, Massachusetts; 3 Division of Geriatric Psychiatry, McLean Hospital, Harvard Medical School, Boston, Massachusetts; 4 Department of Medicine, Massachusetts General Hospital, Harvard Medical School, Boston

## Abstract

**Importance:**

There are limited data on discontinuation rates of antipsychotic medications (APMs) used to treat delirium due to acute hospitalization in the routine care of older adults.

**Objective:**

To investigate discontinuation rates and patient characteristics of APMs used to treat delirium following infection-related hospitalization among older US adults.

**Design, Setting, and Participants:**

This retrospective cohort study was conducted using US claims data (Optum’s deidentified Clinformatics Data Mart database) for January 1, 2004, to May 31, 2022. Patients were aged 65 years or older without prior psychiatric disorders and had newly initiated an APM prescription within 30 days of an infection-related hospitalization. Statistical analysis was performed on December 15, 2022.

**Exposures:**

New use (no prior use any time before cohort entry) of oral haloperidol and atypical APMs (aripiprazole, olanzapine, quetiapine, risperidone, etc).

**Main Outcomes and Measures:**

The primary outcome was APM discontinuation, defined as a gap of more than 15 days following the end of an APM dispensing. Survival analyses and Kaplan-Meier analyses were used.

**Results:**

Our study population included 5835 patients. Of these individuals, 790 (13.5%) were new haloperidol users (mean [SD] age, 81.5 [6.7] years; 422 women [53.4%]) and 5045 (86.5%) were new atypical APM users (mean [SD] age, 79.8 [7.0] years; 2636 women [52.2%]). The cumulative incidence of discontinuation by 30 days after initiation was 11.4% (95% CI, 10.4%-12.3%) among atypical APM users and 52.1% (95% CI, 48.2%-55.7%) among haloperidol users (*P* < .001 for difference between haloperidol vs atypical APMs). We observed an increasing trend in discontinuation rates from 2004 to 2022 (5% increase [95% CI, 3%-7%] per year) for haloperidol users (adjusted hazard ratio, 1.05 [1.03-1.07]; *P* < .001) but not for atypical APM users (1.00 [0.99-1.01]; *P* = .67). Prolonged hospitalization and dementia were inversely associated with the discontinuation of haloperidol and atypical APMs.

**Conclusions and Relevance:**

The findings of this cohort study suggest that the discontinuation rate of newly initiated APMs for delirium following infection-related hospitalization was lower in atypical APM users than in haloperidol users, with prolonged hospitalization and dementia as major associated variables. The discontinuation rate was substantially higher in recent years for haloperidol but not for atypical APMs.

## Key Points

**Question:**

What are the rates and associated patient characteristics of discontinuation of antipsychotic medications (APMs) among older adults following infection-related hospitalization?

**Findings:**

In this cohort study of 5835 patients in the US, we observed discontinuation rates of only 11% for new atypical APM users and 52% for new haloperidol users by 30 days after initiation following infection-related hospitalization. Dementia and prolonged hospitalization were inversely associated with haloperidol and atypical APM discontinuation.

**Meaning:**

These findings suggest that contrary to clinical recommendations, APM discontinuation rates following infection-related hospitalization are low and are lower for atypical APMs than for haloperidol.

## Teaser

This cohort study assesses antipsychotic medication use among older US adults, including rates of and patient characteristics associated with discontinuation, following infection-related hospitalization.

## Introduction

Delirium, which is characterized by acute onset of disturbance of consciousness and cognition,^1^ represents a major burden to the health care system.^2^ It is often associated with serious adverse events such as increased mortality, prolonged length of stay, and functional decline.^3^ Delirium is a common presentation (30%-45%) in older adults hospitalized for infection such as influenza, pneumonia, urinary tract infection, and COVID-19.^4,5,6^ Although antipsychotic medications (APMs) are commonly prescribed to manage behavioral disturbances caused by delirium, these agents are associated with multiple serious adverse clinical outcomes, including death, cardiac arrhythmia, orthostatic hypotension, pneumonia, and urinary dysfunction.^7,8,9^ Therefore, clinical consensus recommends that APMs should be used with caution in older adults and should be discontinued as soon as possible.^10,11,12^ However, there are very limited data on APM discontinuation rates in routine care after delirium due to acute hospitalization. Also, little is known about the factors associated with discontinuation of APMs used for delirium. We aimed to assess discontinuation rates of APMs in older US adults following hospitalization for infection with delirium in routine care. We also sought to assess patient characteristics associated with discontinuation of APMs newly initiated after infection-related hospitalization.

## Methods

### Data Source

For this cohort study, we used Optum’s deidentified Clinformatics Data Mart (CDM) database^13^ claims data for January 1, 2004, to May 31, 2022. The CDM data are derived from a database of administrative health claims for members of large commercial and Medicare Advantage health plans. The CDM database includes more than 62 million unique individuals, spanning all 50 US states and Washington, DC. Based on verified, adjudicated, and deidentified medical and pharmacy claims data, the CDM provides information on patient demographics, enrollment start and end dates, medical diagnoses, dispensed medications, performed procedures, and information related to health care costs and resource utilization. The Mass General Brigham Institutional Review Board approved the study protocol and the waiver of informed consent because this was a secondary use of preexisting deidentified data with a minimal risk of harm to study participants. Our study followed the Strengthening the Reporting of Observational Studies in Epidemiology (STROBE) reporting guideline.

### Study Population

The study cohort consisted of individuals aged 65 years and older with at least 180 days of continuous baseline medical and drug enrollment preceding the index date, allowing gaps of up to 31 days. Patients with a new dispensing of an oral APM (generic names are provided in eTable 1 in Supplement 1) within 30 days of hospital discharge and with any eligible inpatient infection diagnosis were included. New dispensing was defined as no APM use at any time before cohort entry, and the APM dispensing date was the cohort entry date (CED). A prior study showed that new initiation of APMs in a hospital setting is a good proxy for the presence of delirium when cross-validated against the criterion standard delirium diagnosis established by clinical assessment (positive predictive value = 92.0%).^14,15^ To ensure that patients have an adequate medication supply until the first postdischarge follow-up appointment, the discharging clinician typically provides the prescription of a new medication that results from hospitalization.^16,17^ Although delirium is undercoded in administrative databases, it constitutes a majority of indications for APM initiation in the hospital.^18,19^ Since claims data do not contain information on inpatient medication use, we assumed that the new dispensing of an APM in the outpatient setting within 30 days of hospital discharge was a new use for delirium attributable to the infection-related hospitalization (study design diagram in eFigure 2 in Supplement 1). Patients with prior use of or chronic indications for APMs, including schizophrenia and other psychotic disorders, bipolar disorder, and depression at any time before cohort entry, were excluded (using primary or secondary diagnoses in all settings; exclusion criteria and definitions are provided in eTable 2 in Supplement 1) because it is possible that APM use in the index hospitalization for these patients was for the chronic indications.^20,21^ We also excluded patients discharged to a short-term skilled nursing facility (SNF) within 30 days of the APM dispensing date due to the lack of medication use data during SNF stays to determine if the patients were chronic users of APMs. The eligible infection types included COVID-19, influenza, pneumonia, urinary tract infection, endocarditis, soft tissue infection, osteomyelitis, septic arthritis, central nervous system infection, intra-abdominal infection, and bacteremia (using primary or secondary discharge diagnoses; definitions are provided in eTable 3 in Supplement 1). The covariate assessment period (CAP) was defined as the 180 days before (including) the CED. Sensitivity analyses were performed by changing the CAP length to 365 days.

### Discontinuation Assessment

Discontinuation was defined as a gap of more than 15 days (primary analysis) following the end of a prescription dispensing, and we assessed the discontinuation rate since the first APM dispensing day (CED) following the index hospitalization. We censored patients on the earliest occurrence of death, disenrollment from insurance coverage, hospitalization or SNF stay, 1 year after the index date, or chronic indications for APMs. Patients who were censored within 15 days after CED were excluded from the primary analysis because they did not have sufficient follow-up to assess the discontinuation rate with the definition of having a dispensing gap of more than 15 days. We conducted sensitivity analyses using gaps of more than 7 and 30 days to define APM discontinuation. While atypical APMs consist of various agents (eTable 1 in Supplement 1), we focused on haloperidol as the typical APM in the primary analysis because typical APMs other than haloperidol are commonly prescribed to treat nondelirium-related conditions (eg, nausea and vomiting).^22,23^ The percentage of discontinuation at 30, 60, 90, and 180 days following the CED was assessed. We also assessed discontinuation rates in the most commonly used atypical APMs, including aripiprazole, risperidone, quetiapine, and olanzapine specifically. We added days’ supply of the same length to the end of drug exposure, and we capped such an extension at 30 days to avoid overcorrection (eg, if the allowable gap was 15 days, we added a 15-day extended period to the last APM dispensing). This is because the allowable gap could approximate the number (days’ supply length) of leftover pills a patient may have to bridge the prescription gaps.

### Covariates

In the CAP, we assessed baseline covariates including demographic factors (age, sex, and race and ethnicity), baseline conditions (dementia, diabetes, chronic kidney disease, cancer, liver disease, stroke, end-stage kidney disease, anemia, etc), frailty^24^ (measured using a claims-based frailty index validated against clinical measures of frailty^25,26,27,28^), health care utilization (emergency department visits and hospital stays), and calendar year (eTable 4 in Supplement 1 presents all covariates and definitions). Race and ethnicity data were obtained using an algorithm for administrative claims data sets^29^ and are reported as Black, White, other (Asian, Hispanic, or unknown race and ethnicity), or missing. Definitions of the covariates were drawn from published studies (references in eTables 3 and 4 in Supplement 1) and then verified by 2 board-certified physicians (J.M.W. and K.J.L.).

### Statistical Analysis

We compared patient characteristics of haloperidol vs APM users by computing the difference in the prevalence or mean of each factor with its 95% CI. To estimate discontinuation accounting for censoring, we conducted survival analyses stratified by haloperidol vs atypical APMs. Kaplan-Meier analyses were performed to study treatment discontinuation at each time point (30, 60, 90, 180, and 365 days after CED). We assessed the association of various risk factors as the hazard ratio of APM discontinuation, using Cox proportional hazards regression and adjusting for the aforementioned covariates. Since claims data do not provide medication use information for patients during short-term SNF or hospitalization stays, the SNF or hospital stay can potentially lead to misclassification of APM discontinuation. Therefore, in the primary analysis, we censored patients upon SNF or hospital admission during follow-up. We tested the robustness of our results in a sensitivity analysis without such censoring. We used 2 methods to account for competing risks due to death. In the primary analysis, we applied inverse probability of censoring weighting (IPCW), with the weights being the inverse probability of censoring due to death, estimated by logistic regression conditioning on baseline covariates. In the secondary analysis, we used a Fine and Gray model to estimate discontinuation rates after accounting for the competing risk due to death.^30,31^ A 2-sided *P* value of <.05 was used to indicate statistical significance. All analyses were conducted using the Aetion Evidence Platform^32^ (Aetion Inc) and R, version 4.2.1 (R Project for Statistical Computing). Statistical analysis was performed on December 15, 2022.

## Results

### Patient Characteristics

This cohort study included 5835 patients in the primary analysis. Of these individuals, 790 (13.5%) were new haloperidol users (mean [SD] age, 81.5 [6.7] years; 422 women [53.4%] and 368 men [46.6%]) and 5045 (86.5%) were new atypical APM users (mean [SD] age, 79.8 [7.0] years; 2636 women [52.2%] and 2409 men [47.8%]). The cohort formation process is provided in eFigure 1 in Supplement 1. For haloperidol vs atypical APM users, race and ethnicity were reported as Black (117 [14.8%] vs 703 [13.9%]), White (552 [69.9%] vs 3406 [67.5%]), or other race or ethnicity or missing (121 [15.3%] vs 936 [18.6%]). Based on unadjusted prevalence, haloperidol users were older and more likely to have bacteremia, cancer, heart failure, gastrointestinal bleeding, anemia, liver disease, or end-stage kidney disease compared with atypical users but were less likely to be of Black or other race or ethnicity, to be mildly frail, or to have dementia or COVID-19 (Table 1).

**Table 1.** Characteristics of Patients Receiving Antipsychotic Medications Within 30 Days of Hospitalization for COVID-19 and Other Infections

Characteristic	No. of patients (%) (N = 5835)		Simple difference, % (95% CI)^a^
	Haloperidol users (n = 790)	Atypical APM users (n = 5045)	
Age, y			
65-74	136 (17.2)	1277 (25.3)	−8.1 (−11.0 to −5.2)
75-84	319 (40.4)	2132 (42.3)	−1.9 (−5.6 to 1.8)
≥85	335 (42.4)	1636 (32.4)	10.0 (6.3 to 13.7)
Sex			
Male	368 (46.6)	2409 (47.8)	−1.2 (−4.9 to 2.6)
Female	422 (53.4)	2636 (52.2)	1.2 (−2.6 to 4.9)
Race and ethnicity			
Black	117 (14.8)	703 (13.9)	0.9 (−1.8 to 3.5)
White	552 (69.9)	3406 (67.5)	2.4 (−1.1 to 5.8)
Other or missing^b^	121 (15.3)	936 (18.6)	−3.2 (−6.0 to −0.5)
Frailty score			
Robust	14 (1.8)	90 (1.8)	0.0 (−1.0 to 1.0)
Prefrail	298 (37.7)	1811 (35.9)	1.8 (−1.8 to 5.5)
Mildly frail	359 (45.4)	2483 (49.2)	−3.8 (−7.5 to 0.0)
Moderate to severely frail	119 (15.1)	661 (13.1)	2.0 (−0.7 to 4.6)
Infection type during hospitalization^c^			
COVID-19	30 (3.8)	369 (7.3)	−3.5 (−5.0 to −2.0)
Influenza	<11 (0.6)	55 (1.1)	−0.5 (−1.1 to 0.2)
Urinary tract	359 (45.4)	2361 (46.8)	−1.4 (−5.1 to 2.4)
Pneumonia	308 (39.0)	1911 (37.9)	1.1 (−2.5 to 4.8)
Bacteremia	85 (10.8)	417 (8.3)	2.5 (0.2 to 4.8)
Endocarditis	<11 (0.3)	32 (0.6)	−0.4 (−0.8 to 0.0)
Soft tissue	93 (11.8)	481 (9.5)	2.2 (−0.2 to 4.6)
Osteomyelitis/arthritis	<11 (0.4)	44 (0.9)	−0.5 (−1.0 to 0.0)
Central nervous system	14 (1.8)	50 (1.0)	0.8 (−0.2 to 1.7)
Intra-abdominal and peritonitis	17 (2.2)	78 (1.5)	0.6 (−0.5 to 1.7)
Comorbidity			
Dementia	422 (53.4)	2928 (58.0)	−4.6 (−8.4 to −0.9)
Heart failure	337 (42.7)	1813 (35.9)	6.7 (3.0 to 10.4)
Hypertension	350 (44.3)	2139 (42.4)	1.9 (−1.8 to 5.6)
Ischemic heart disease	663 (83.9)	4360 (86.4)	−2.5 (−5.2 to 0.2)
Falls	167 (21.1)	1085 (21.5)	−0.4 (−3.4 to 2.7)
Chronic kidney disease	280 (35.4)	1646 (32.6)	2.8 (−0.8 to 6.4)
Cancer	150 (19.0)	781 (15.5)	3.5 (0.6 to 6.4)
Deep vein thrombosis	18 (2.3)	111 (2.2)	0.1 (−1.0 to 1.2)
Anemia	392 (49.6)	2226 (44.1)	5.5 (1.8 to 9.2)
Atrial fibrillation	282 (35.7)	1642 (32.5)	3.1 (−0.4 to 6.7)
Liver disease	118 (14.9)	610 (12.1)	2.8 (0.2 to 5.5)
Stroke	186 (23.5)	1322 (26.2)	−2.7 (−5.9 to 0.5)
End-stage kidney disease	47 (5.9)	208 (4.1)	1.8 (0.1 to 3.6)
Gastrointestinal bleeding	97 (12.3)	435 (8.6)	3.7 (1.2 to 6.1)
Alcohol abuse or dependence	42 (5.3)	291 (5.8)	−0.5 (−2.1 to 1.2)
Diabetes	298 (37.7)	1918 (38.0)	−0.3 (−3.9 to 3.3)
Health care utilization in the 180 d before cohort entry			
Emergency department visit^d^	666 (84.3)	4155 (82.4)	1.9 (−0.8 to 4.7)
Hospitalization, d^e^			
≤7	283 (35.8)	1885 (37.4)	−1.5 (−5.1 to 2.1)
8-30	391 (49.5)	2389 (47.4)	2.1 (−1.6 to 5.9)
>30	116 (14.7)	771 (15.3)	−0.6 (−3.3 to 2.1)

Abbreviation: APM, antipsychotic medication.

^a^ Simple difference of 2 proportions.

^b^ Other indicates Asian, Hispanic, or unknown race and ethnicity. Data were missing or unknown for 356 patients.

^c^ Defined as any inpatient confinement of eligible infection within 30 days of the cohort entry date.

^d^ Defined as any emergency department visit during the covariate assessment period.

^e^ Defined as the total number of days inpatient confinement occurred during the covariate assessment period.

### Discontinuation Assessment

The IPCW-adjusted cumulative incidence of discontinuation by 30 days was 11.4% (95% CI, 10.4%-12.3%) among atypical APM new users, with rates of 53.7% (52.1%-55.2%), 64.1% (62.5%-65.6%), and 76.3% (74.7%-77.7%) at 60, 90, and 180 days, respectively. The corresponding discontinuation rate was 52.1% (95% CI, 48.2%-55.7%), 78.8% (75.1%-81.9%), 85.0% (81.5%-87.9%), and 93.7% (90.4%-95.9%) by 30, 60, 90, and 180 days for haloperidol users, respectively (Table 2). Patients with a new prescription of haloperidol had a higher discontinuation rate compared with atypical APM users (93.7% [95% CI, 90.4%-95.9%] vs 76.3% [74.7%-77.7%] by 180 days; log-rank test *P* < .001, proportional hazards assumptions were met by visual assessment of Kaplan-Meier curves) (Figure). Among atypical APM users, discontinuation rates were comparable across users of aripiprazole, risperidone, quetiapine, and olanzapine. The estimated cumulative incidence of discontinuation based on the Fine and Gray competing risk model was similar to that based on primary analysis (Table 2). We observed an increasing trend in discontinuation rates from 2004 to 2022 (5% increase [95% CI, 3%-7%] per year) for haloperidol users (adjusted hazard ratio [aHR], 1.05 [1.03-1.07]; *P* < .001) but not for atypical APM users (1.00 [0.99-1.01]; *P* = .67; Table 3).

**Table 2.** Antipsychotic Medication Discontinuation Rate After Initiation for Infection-Related Hospitalization

Medication	Discontinuation rate, % (95% CI)		
	Crude	IPW adjusted	Fine and Gray adjusted^a^
Haloperidol, d^b^			
30	51.4 (47.5 to 55.0)	52.1 (48.2 to 55.7)	48.5 (48.4 to 48.6)
60	78.6 (74.9 to 81.7)	78.8 (75.1 to 81.9)	70.5 (70.4 to 70.5)
90	84.8 (81.3 to 87.7)	85.0 (81.5 to 87.9)	75.2 (75.2 to 75.3)
180	93.6 (90.2 to 95.8)	93.7 (90.4 to 95.9)	81.5 (81.4 to 81.5)
365	96.2 (92.5 to 98.1)	96.4 (92.7 to 98.2)	82.9 (82.8 to 82.9)
All atypical APMs, d			
30	11.3 (10.4 to 12.2)	11.4 (10.4 to 12.3)	11.0 (11.0 to 11.0)
60	53.5 (52.0 to 55.1)	53.7 (52.1 to 55.2)	50.5 (50.5 to 50.5)
90	64.0 (62.4 to 65.5)	64.1 (62.5 to 65.6)	59.9 (59.9 to 59.9)
180	76.2 (74.6 to 77.6)	76.3 (74.7 to 77.7)	70.7 (70.7 to 70.7)
365	84.6 (83.0 to 86.0)	84.6 (83.1 to 86.1)	77.9 (77.9 to 77.9)
Specific atypical APM, d			
Aripiprazole			
30	12.9 (3.5 to 21.4)	13.1 (3.5 to 21.6)	12.5 (12.1 to 12.9)
60	57.7 (41.0 to 69.6)	57.9 (41.1 to 69.8)	55.6 (54.6 to 56.6)
90	67.6 (50.5 to 78.8)	67.8 (50.7 to 78.9)	65.2 (64.2 to 66.2)
180	77.3 (59.2 to 87.4)	77.4 (59.3 to 87.4)	74.6 (73.6 to 75.6)
365	94.0 (64.9 to 99.0)	94.0 (64.9 to 99.0)	90.6 (89.6 to 91.6)
Olanzapine			
30	15.4 (12.6 to 18.2)	15.5 (12.6 to 18.2)	14.8 (14.7 to 14.8)
60	57.9 (53.6 to 61.8)	57.9 (53.6 to 61.8)	53.0 (52.9 to 53.0)
90	70.1 (65.9 to 73.8)	70.0 (65.7 to 73.7)	63.5 (63.5 to 63.6)
180	81.2 (77.1 to 84.6)	81.2 (77.1 to 84.6)	72.7 (72.7 to 72.8)
365	89.9 (85.6 to 92.9)	89.8 (85.5 to 92.9)	79.3 (79.2 to 79.3)
Quetiapine			
30	10.6 (9.5 to 11.7)	10.7 (9.5 to 11.8)	10.4 (10.4 to 10.4)
60	53.3 (51.3 to 55.2)	53.3 (51.4 to 55.3)	50.5 (50.5 to 50.5)
90	63.7 (61.7 to 65.6)	63.8 (61.8 to 65.7)	60.1 (60.1 to 60.1)
180	75.7 (73.7 to 77.5)	75.7 (73.7 to 77.5)	70.8 (70.8 to 70.8)
365	83.4 (81.4 to 85.2)	83.4 (81.4 to 85.2)	77.5 (77.5 to 77.5)
Risperidone			
30	10.0 (8.0 to 12.0)	10.1 (8.1 to 12.1)	9.8 (9.8 to 9.8)
60	50.7 (47.1 to 54.1)	50.9 (47.3 to 54.3)	47.9 (47.8 to 47.9)
90	60.4 (56.7 to 63.7)	60.4 (56.8 to 63.8)	56.6 (56.5 to 56.7)
180	73.7 (70.0 to 76.9)	73.7 (70.1 to 77.0)	68.3 (68.2 to 68.4)
365	83.7 (80.0 to 86.7)	83.7 (79.9 to 86.7)	76.8 (76.8 to 76.9)
Other atypical			
30	16.2 (7.3 to 24.3)	16.5 (7.4 to 24.7)	16.1 (15.7 to 16.4)
60	58.0 (43.6 to 68.7)	58.4 (44.0 to 69.0)	55.0 (54.3 to 55.8)
90	60.6 (45.7 to 71.4)	61.0 (46.2 to 71.8)	57.3 (56.5 to 58.0)
180	82.5 (63.8 to 91.5)	82.5 (63.9 to 91.5)	75.7 (74.9 to 76.6)
365	91.3 (70.4 to 97.4)	91.4 (70.7 to 97.5)	83.1 (82.3 to 83.9)

Abbreviations: APM, antipsychotic medication; IPW, inverse probability weighting.

^a^ Fine and Gray proportional subdistribution hazard model.

^b^ Time was defined as the number of days after cohort entry (including the number of days required to start follow-up).

**Figure.**
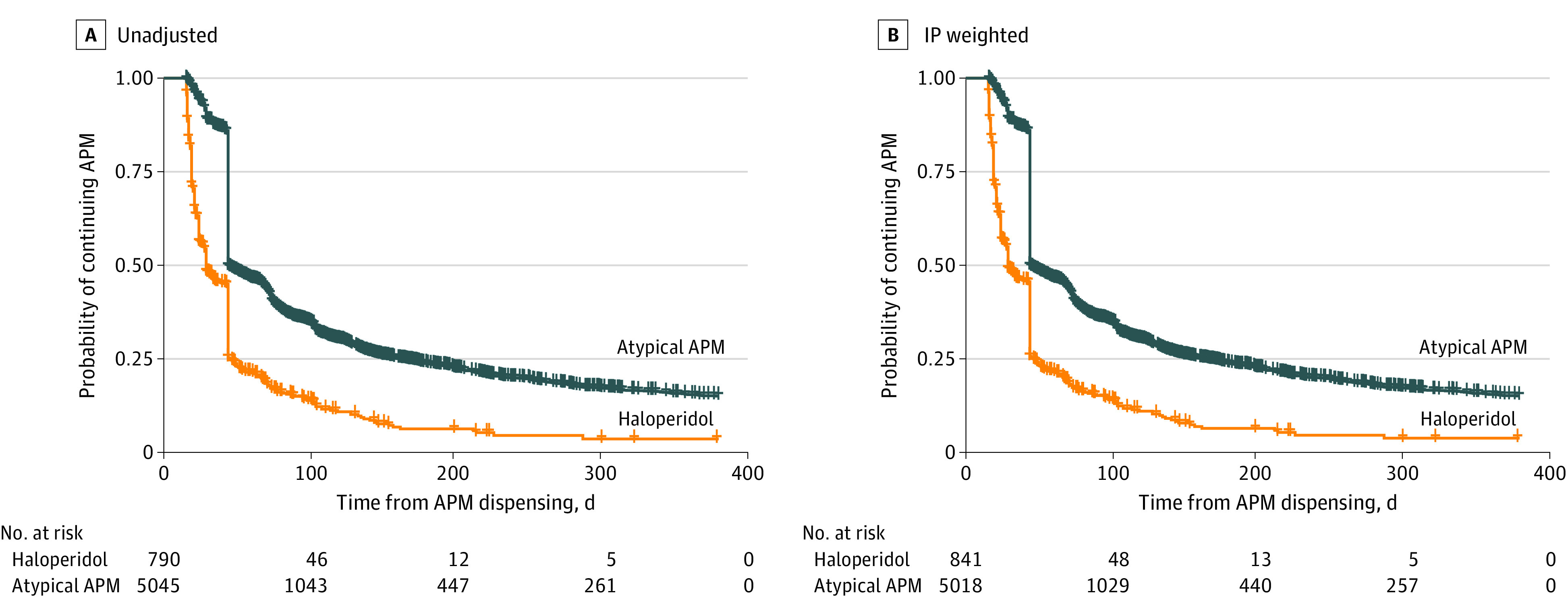
Kaplan-Meier Curves of Antipsychotic Medication (APM) Discontinuation Among Older Adults After Initiation for Infection-Related Hospitalization A, Unadjusted curves. B, Inverse probability–weighted curves. Crosses indicate censoring times. APM indicates antipsychotic medication.

**Table 3.** Inverse Probability of Censoring Weight-Adjusted Hazard Ratios of Antipsychotic Medication Discontinuation After Initiation for Infection-Related Hospitalization^a^

Characteristic	Adjusted HR (95% CI)^b^	
	Haloperidol	Atypical antipsychotic medication
Age, y		
65-74	1 [Reference]	1 [Reference]
75-84	1.04 (0.82-1.33)	0.97 (0.90-1.06)
≥85	1.23 (0.96-1.56)	1.02 (0.92-1.12)
Sex		
Female	1 [Reference]	1 [Reference]
Male	0.85 (0.72-1.00)	1.06 (0.99-1.14)
Race and ethnicity		
Black	1.08 (0.87-1.33)	1.02 (0.92-1.12)
White	1 [Reference]	1 [Reference]
Other or missing^c^	1.08 (0.85-1.36)	1.06 (0.98-1.15)
Frailty score		
Robust	1 [Reference]	1 [Reference]
Prefrail	0.88 (0.53-1.46)	0.98 (0.79-1.20)
Mildly frail	0.75 (0.44-1.28)	0.92 (0.74-1.15)
Moderate to severely frail	0.67 (0.36-1.23)	0.91 (0.72-1.16)
Infection type (reason for admission)		
COVID-19	1.34 (0.85-2.10)	1.10 (0.96-1.26)
Influenza	0.80 (0.44-1.46)	0.94 (0.67-1.33)
Urinary tract	1.01 (0.78-1.31)	0.95 (0.85-1.06)
Pneumonia	1.47 (1.14-1.90)	1.12 (1.01-1.24)
Bacteremia	0.99 (0.74-1.33)	1.05 (0.93-1.19)
Endocarditis	0.57 (0.12-2.65)	1.19 (0.91-1.56)
Soft tissue	1.26 (0.95-1.69)	0.98 (0.86-1.12)
Osteomyelitis/septic arthritis	1.22 (0.72-2.07)	1.32 (1.00-1.75)
Central nervous system	0.83 (0.38-1.81)	1.04 (0.72-1.49)
Intra-abdominal and peritonitis	1.47 (0.88-2.43)	1.26 (0.97-1.64)
Comorbidity		
Dementia	0.71 (0.58-0.87)	0.80 (0.74-0.86)
Heart failure	1.29 (1.07-1.57)	1.03 (0.95-1.11)
Hypertension	0.95 (0.79-1.14)	1.03 (0.96-1.11)
Ischemic heart disease	1.07 (0.87-1.34)	0.90 (0.82-1.00)
Falls	0.94 (0.75-1.18)	1.00 (0.92-1.08)
Chronic kidney disease	0.92 (0.76-1.11)	1.07 (0.99-1.15)
Cancer	1.12 (0.90-1.40)	1.12 (1.03-1.23)
Deep vein thrombosis	0.85 (0.37-1.98)	1.12 (0.93-1.35)
Anemia	0.93 (0.78-1.12)	0.97 (0.91-1.05)
Atrial fibrillation	1.02 (0.85-1.23)	1.04 (0.96-1.11)
Liver disease	1.02 (0.80-1.30)	1.10 (0.99-1.23)
Stroke	1.18 (0.96-1.45)	0.89 (0.82-0.97)
End-stage kidney disease	1.09 (0.70-1.69)	0.89 (0.75-1.06)
Gastrointestinal bleeding	1.46 (1.14-1.87)	0.96 (0.85-1.08)
Alcohol abuse or dependence	1.20 (0.82-1.73)	0.88 (0.76-1.02)
Diabetes	0.77 (0.65-0.92)	1.02 (0.95-1.09)
Health care utilization in the 180 d before cohort entry		
Emergency department visit	1.13 (0.89-1.43)	0.98 (0.91-1.07)
Hospitalization, d		
≤7	1 [Reference]	1 [Reference]
8-30	0.78 (0.65-0.93)	0.96 (0.89-1.03)
>30	0.61 (0.45-0.84)	0.86 (0.77-0.97)
Cohort entry year	1.05 (1.03-1.07)	1.00 (0.99-1.01)

Abbreviation: HR, hazard ratio.

^a^ The inverse probability of censoring weight is the inverse probability of censoring due to death, estimated by logistic regression conditioning on baseline covariates.

^b^ Adjusted for all covariates listed here. Comparison for all dichotomous variables was between presence vs absence. Cohort entry year was treated as a continuous variable.

^c^ Other indicates Asian, Hispanic, or unknown race and ethnicity. Data were missing or unknown for 356 patients.

### Patient Characteristics Associated With Discontinuation

Based on the IPCW-adjusted models, factors associated with discontinuation of haloperidol included pneumonia (aHR, 1.47 [95% CI, 1.14-1.90]), heart failure (aHR, 1.29 [95% CI, 1.07-1.57]), and gastrointestinal bleeding (aHR, 1.46 [95% CI, 1.14-1.87]). Factors associated with discontinuation of atypical APMs included pneumonia (aHR, 1.12 [95% CI, 1.01-1.24]), cancer (aHR, 1.12 [95% CI, 1.03-1.23]), and osteomyelitis/septic arthritis (aHR, 1.32 [95% CI, 1.00-1.75]). In contrast, prolonged hospitalization and dementia were inversely associated with discontinuation of haloperidol and atypical APMs when comparing inpatient stays of more than 30 days to less than 7 days (aHR, 0.61 [95% CI, 0.45-0.84] vs aHR, 0.86 [95% CI, 0.77-0.97] for haloperidol vs atypical APM users) and patients with vs without dementia (aHR, 0.71 [95% CI, 0.58-0.87] vs aHR, 0.80 [95% CI, 0.74-0.86] for haloperidol vs atypical APM users). Patients with baseline diabetes were less likely to discontinue haloperidol (aHR, 0.77 [95% CI, 0.65-0.92]), while patients with baseline stroke (aHR, 0.89 [95% CI, 0.82-0.97]) or ischemic heart disease (aHR, 0.90 [95% CI, 0.82-1.00]) were less likely to discontinue atypical APMs (Table 3).

### Sensitivity Analysis

Sensitivity analyses varying the definition of APM discontinuation as having a dispensing gap of more than 7 and 30 days revealed similar patterns of discontinuation rates and associations with the covariates as the primary analyses (eTables 5-8 in Supplement 1), although there was a noticeable trend in which using a larger allowable gap to define APM discontinuation yielded lower discontinuation rates. Changing the CAP length to 365 days also revealed similar patterns of discontinuation rates and associations (eTables 9 and 10 in Supplement 1). Sensitivity analyses with no SNF or hospitalization censoring yielded similar results (eTables 11 and 12 in Supplement 1).

## Discussion

This cohort study used data from a large US national commercial claims database to evaluate APM discontinuation rates among older adults following infection-related hospitalization. For new atypical APM users vs new haloperidol users, we observed that only 11.4% vs 52.1% discontinued the mediation by 30 days and 76.3% vs 93.7% discontinued it by 180 days. These findings suggest that prolonged hospitalization and dementia were inversely associated with both haloperidol and atypical APM discontinuation. There was a notable trend of increased discontinuation in the later years for haloperidol but not for atypical APMs.

There are very limited data in the literature about APM prescribing and discontinuation for delirium. Prior studies reported that approximately 30% of patients who newly initiate treatment with an APM during hospitalization are discharged with the medication.^19,33^ In this study, we consistently observed that APM discontinuation following acute hospitalization did not occur in a timely fashion. Clinicians may be reluctant to actively discontinue the ongoing treatment after the patient’s condition is stabilized, which may explain the low rates of APM discontinuation after delirium onset. These findings call for further investigation of potential modifiable risk factors of prolonged APM use and proactive interventions to facilitate discontinuation of these potentially inappropriate medications in older adults.^10,11,12^ Commonly used interventions include patient education, clinician education (eg, continuing medical education courses), use of tapering or deprescribing plans guided by health care professionals, and implementation of monitoring protocols.^34^ Specific strategies to support behavioral change include adding visual cues to the environment (eg, deprescribing algorithms, medication checklists, etc) and building prompts into routine workflow (eg, electronic health record alerts, reminder letters or messages, etc).^35^

We observed a consistent trend that patients with a new prescription of a typical APM (ie, haloperidol) had a higher discontinuation rate compared with atypical APM users. This is contrary to clinical recommendations to discontinue both types of APMs as soon as the delirious state or acute behavioral disturbance has resolved.^10,11,12^ There are conflicting data in the literature about the safety of typical vs atypical APMs in older adults. Prior studies have reported a higher risk of adverse effects (eg, extrapyramidal effects) for haloperidol compared with atypical APMs.^36,37,38^ While some studies reported a higher risk of death with typical APMs compared with atypical APMs, others suggested that the risk of serious adverse events (eg, death or cardiac arrythmias) were comparable in typical vs atypical APM users.^39,40,41^ After synthesizing the available data, the US Food and Drug Administration (FDA) issued a black box warning for the use of all APMs in the treatment of behavioral symptoms for older adults with dementia.^42^ Our findings suggest that haloperidol users who entered the study cohort in later years had a notably higher probability of APM discontinuation. This may be explained by an increasing awareness of the higher side-effect profile of typical APMs after the FDA black box warning.^42^ In contrast, we did not observe such a time trend for atypical APM discontinuation, suggesting less awareness of the potential harms associated with prolonged use of atypical APMs in older adults or perceived safer profiles of atypical APMs compared with typical APMs that may not be evidence based.^43,44^

Our findings suggest that prolonged hospitalization was inversely associated with the discontinuation of APMs for delirium, with a dose-response association. Prior studies reported that prolonged hospital or intensive care unit stay is a risk factor for the development of acute delirium.^45,46,47^ This highlights the importance of advancing care and discharging older adults during acute hospitalizations in a timely fashion. We also identified dementia as a risk factor for prolonged APM use after delirium onset. Approximately 46% to 56% of patients with dementia develop delirium after being hospitalized.^48,49^ Although delirium is considered an acute change in mental status, its recovery can be protracted in older adults, especially those with dementia.^50^ A prior study reported that more than 92% of individuals living with dementia remained in a confusional state for more than 90 days after being diagnosed with delirium in the hospital.^51^ A 2017 study reported that among patients with delirium in the palliative care setting, risperidone and haloperidol users experienced worsened delirium compared with those in the placebo group.^52^ Therefore, special attention to potential adverse events associated with prolonged use of APMs is warranted for individuals living with dementia.

### Limitations

There are several limitations of this study. First, our analyses may have unmeasured confounders, such as socioeconomic status, lifestyle patterns, and psychosocial factors, that are not well captured in claims data. Therefore, the associations observed in our study should be viewed as hypothesis generating rather than causal, particularly for the factors with small effect sizes and not consistently associated with APM discontinuation across types of APMs or sensitivity analyses. Second, we used administrative insurance claims data to define our covariates. The accuracy and completeness of the *International Classification of Diseases* codes in claims data may be questionable. Claims data also do not provide information to distinguish regular use from as-needed use, which could lead to misclassification of discontinuation. Third, we used new initiation of APMs following acute hospitalization as the proxy for having delirium. Although this is based on a prior validation study with high positive predictive value (92.0%),^14^ there can still be misclassification (ie, APMs were used for a nondelirium indication). We therefore excluded an extensive list of APM-indicated psychiatric conditions (eTable 2 in Supplement 1) and our findings cannot be generalized to these populations. While it is possible that some APMs were used for indications other than delirium, the discontinuation rates of specific APMs with higher propensity for use for other indications (eg, quetiapine may be more likely to be used for insomnia due to its sedating effect) were not substantially different from that of other atypical APMs (Table 2). We observed that discontinuation rates based on IPCW (adjusted for censoring due to death) were generally higher than those based on the Fine and Gray models, which did not remove death from the risk set, resulting in larger denominators when calculating discontinuation rates. Lastly, APM discontinuation defined by a fixed allowable gap was also subject to misclassification, but our sensitivity analyses using gaps of more than 7 and 30 days to define APM discontinuation yielded similar results. Patients in our cohort were less likely to be prescribed a longer supply due to exclusion of patients with chronic indications for APMs. Requiring a long gap may preclude assessment of early discontinuation following initiation of APMs. Therefore, we did not examine gaps longer than 30 days.

## Conclusions

In conclusion, we found that 52.1% of older adults who newly initiated haloperidol and 11.4% of older adults who newly initiated an atypical APM following acute infection-related hospitalization discontinued these medications by 30 days. Dementia and prolonged hospitalization were inversely associated with discontinuation of haloperidol and atypical APMs. There was a notable time trend suggesting that the discontinuation rate was substantially higher in later years for haloperidol but not for atypical APMs. Given multiple serious adverse reactions associated with APM use, our findings call for effective interventions to proactively discontinue APMs when they are no longer indicated.

## Notes

Supplement 1. **eFigure 1.** Selection of Study Population **eFigure 2.** Study Design Diagram **eTable 1.** List of Antipsychotic Medications **eTable 2.** List of Antipsychotic Medication–Indicated Psychiatric Conditions **eTable 3.** Eligible Infection Conditions for Cohort Inclusion **eTable 4.** List of Covariates **eTable 5.** Sensitivity Analyses of Antipsychotic Medication Discontinuation Rate (Prescription Gap >7 Days) After Initiation for Infection-Related Hospitalization **eTable 6.** Sensitivity Analyses of Antipsychotic Medication Discontinuation Rate (Prescription Gap >30 Days) After Initiation for Infection-Related Hospitalization **eTable 7.** Sensitivity Analyses of Inverse Probability Weight–Adjusted Hazard Ratios of Antipsychotic Medication Discontinuation (Prescription Gap >7 Days) After Initiation for Infection-Related Hospitalization **eTable 8.** Sensitivity Analyses of Inverse Probability Weight–Adjusted Hazard Ratios of Antipsychotic Medication Discontinuation (Prescription Gap >30 Days) After Initiation for Infection-Related Hospitalization **eTable 9.** Sensitivity Analyses of Antipsychotic Medication Discontinuation Rate After Initiation for Infection-Related Hospitalization, With 365 Days of Baseline Enrollment, Covariate Assessment Period, and Washout Period to Define New APM Use **eTable 10.** Sensitivity Analyses of Hazard Ratios of Antipsychotic Medication Discontinuation After Initiation for Infection-Related Hospitalization, With 365 Days of Baseline Enrollment, Covariate Assessment Period, and Washout Period to Define New APM Use **eTable 11.** Sensitivity Analyses of Antipsychotic Medication Discontinuation Rate After Initiation for Infection-Related Hospitalization, Without Censoring for Skilled Nursing Facility/Hospitalization During Follow-up **eTable 12.** Sensitivity Analyses of Hazard Ratios of Antipsychotic Medication Discontinuation After Initiation for Infection-Related Hospitalization, Without Censoring for Skilled Nursing Facility/Hospitalization During Follow-up
eReferences

Supplement 2.
Data Sharing Statement
