# Supplementary material for: Antipsychotic Medication Use Among Older Adults Following Infection-Related Hospitalization
Source: JAMA Netw Open. 2023 Feb 17;6(2):e230063. doi: 10.1001/jamanetworkopen.2023.0063 (PMC9938426; doi:10.1001/jamanetworkopen.2023.0063)
Supplement: Supplement 1. — eFigure 1. Selection of Study Population eFigure 2. Study Design Diagram eTable 1. List of Antipsychotic Medications eTable 2. List of Antipsychotic Medication–Indicated Psychiatric Conditions eTable 3. Eligible Infection Conditions for Cohort Inclusion eTable 4. List of Covariates eTable 5. Sensitivity Analyses of Antipsychotic Medication Discontinuation Rate (Prescription Gap >7 Days) After Initiation for Infection-Related Hospitalization eTable 6. Sensitivity Analyses of Antipsychotic Medication Discontinuation Rate (Prescription Gap >30 Days) After Initiation for Infection-Related Hospitalization eTable 7. Sensitivity Analyses of Inverse Probability Weight–Adjusted Hazard Ratios of Antipsychotic Medication Discontinuation (Prescription Gap >7 Days) After Initiation for Infection-Related Hospitalization eTable 8. Sensitivity Analyses of Inverse Probability Weight–Adjusted Hazard Ratios of Antipsychotic Medication Discontinuation (Prescription Gap >30 Days) After Initiation for Infection-Related Hospitalization eTable 9. Sensitivity Analyses of Antipsychotic Medication Discontinuation Rate After Initiation for Infection-Related Hospitalization, With 365 Days of Baseline Enrollment, Covariate Assessment Period, and Washout Period to Define New APM Use eTable 10. Sensitivity Analyses of Hazard Ratios of Antipsychotic Medication Discontinuation After Initiation for Infection-Related Hospitalization, With 365 Days of Baseline Enrollment, Covariate Assessment Period, and Washout Period to Define New APM Use eTable 11. Sensitivity Analyses of Antipsychotic Medication Discontinuation Rate After Initiation for Infection-Related Hospitalization, Without Censoring for Skilled Nursing Facility/Hospitalization During Follow-up eTable 12. Sensitivity Analyses of Hazard Ratios of Antipsychotic Medication Discontinuation After Initiation for Infection-Related Hospitalization, Without Censoring for Skilled Nursing Facility/Hospitalization During Follow-up eReferences [file jamanetwopen-e230063-s001.pdf]

Supplementary Online Content

Zhang Y, Wilkins JM, Bessette LG, York C, Wong V, Lin KJ. Antipsychotic medication use among older adults following infection-related hospitalization. *JAMA Netw Open*. 2023;6(2):e230063. doi:10.1001/jamanetworkopen.2023.0063

eFigure 1. Selection of Study Population

eFigure 2. Study Design Diagram

eTable 1. List of Antipsychotic Medications

eTable 2. List of Antipsychotic Medication–Indicated Psychiatric Conditions

eTable 3. Eligible Infection Conditions for Cohort Inclusion

eTable 4. List of Covariates

eTable 5. Sensitivity Analyses of Antipsychotic Medication Discontinuation Rate (Prescription Gap >7 Days) After Initiation for Infection-Related Hospitalization

eTable 6. Sensitivity Analyses of Antipsychotic Medication Discontinuation Rate (Prescription Gap >30 Days) After Initiation for Infection-Related Hospitalization

eTable 7. Sensitivity Analyses of Inverse Probability Weight–Adjusted Hazard Ratios of Antipsychotic Medication Discontinuation (Prescription Gap >7 Days) After Initiation for Infection-Related Hospitalization

eTable 8. Sensitivity Analyses of Inverse Probability Weight–Adjusted Hazard Ratios of Antipsychotic Medication Discontinuation (Prescription Gap >30 Days) After Initiation for Infection-Related Hospitalization

eTable 9. Sensitivity Analyses of Antipsychotic Medication Discontinuation Rate After Initiation for Infection-Related Hospitalization, With 365 Days of Baseline Enrollment, Covariate Assessment Period, and Washout Period to Define New APM Use

eTable 10. Sensitivity Analyses of Hazard Ratios of Antipsychotic Medication Discontinuation After Initiation for Infection-Related Hospitalization, With 365 Days of Baseline Enrollment, Covariate Assessment Period, and Washout Period to Define New APM Use

eTable 11. Sensitivity Analyses of Antipsychotic Medication Discontinuation Rate After Initiation for Infection-Related Hospitalization, Without Censoring for Skilled Nursing Facility/Hospitalization During Follow-up

eTable 12. Sensitivity Analyses of Hazard Ratios of Antipsychotic Medication Discontinuation After Initiation for Infection-Related Hospitalization, Without Censoring for Skilled Nursing Facility/Hospitalization During Follow-up

eReferences

This supplementary material has been provided by the authors to give readers additional information about their work.

© 2023 Zhang Y et al. *JAMA Network Open*.

eFigure 1. Selection of Study Population

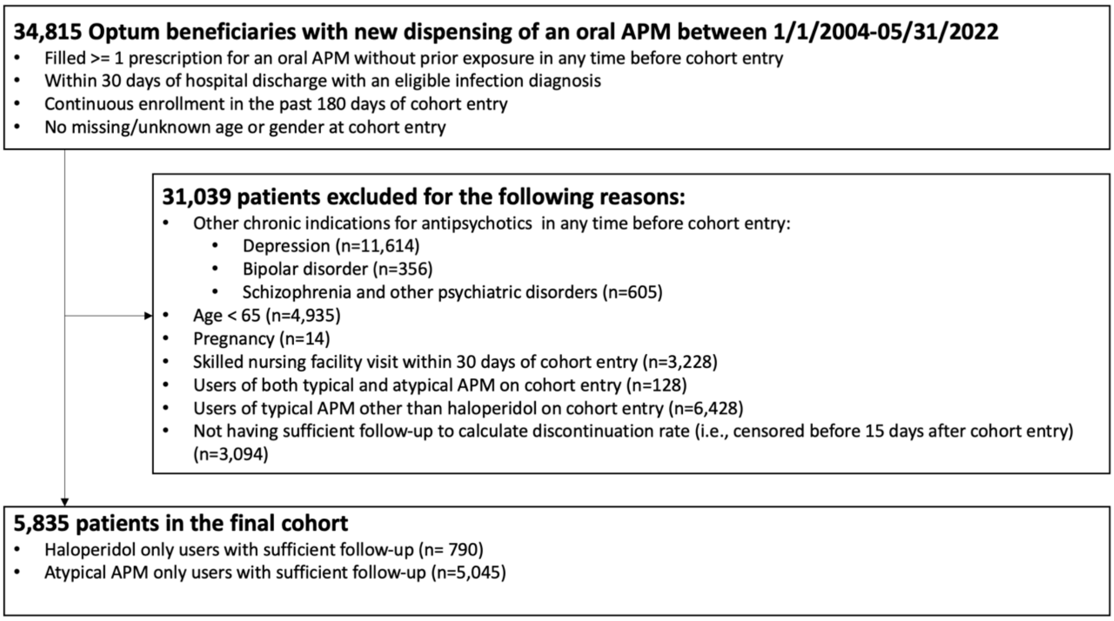

© 2023 Zhang Y et al. *JAMA Network Open*.

eFigure 2. Study Design Diagram

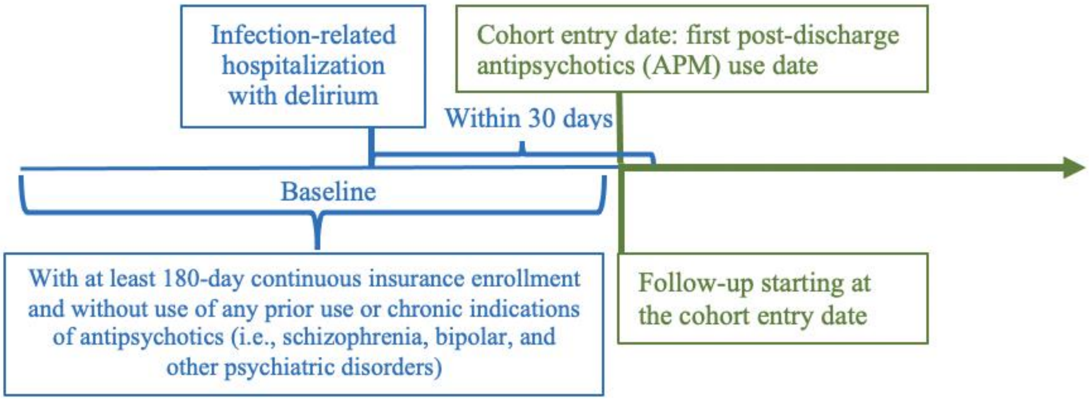

© 2023 Zhang Y et al. *JAMA Network Open*.

eTable 1. List of Antipsychotic Medications

Oral Typical antipsychotics	Oral Atypical antipsychotics
Chlorpromazine	Aripiprazole
Droperidol	Asenapine
Fluphenazine	Clozapine
Loxapine	Iloperidone
Perphenazine	Lurasidone
Pimozide	Paliperidone
Prochlorperazine	Risperidone
Thiothixene	Olanzapine
Thioridazine	Quetiapine
Trifluoperazine	Ziprasidone
Haloperidol	Amisulpride
	Brexpiprazole
	Cariprazine
	Pimavanserin

© 2023 Zhang Y et al. *JAMA Network Open*.

eTable 2. List of Antipsychotic Medication–Indicated Psychiatric Conditions

**APM-indicated psychiatric conditions**	**Codes/Definition**
Schizophrenia and other psychotic disorders	Diagnosis Code, ICD-9 is any of: { “295.20”, “295.30”, “295.70”, “297.00”, “297.10”, “298.30”, “298.40”, “298.80”, “298.90”, “307.2”, “295.10”, “295.40”, “295.60”, “295.80”, “295.90”, “297.2”, “297.30”, “307.23” }<br>Diagnosis Code, ICD-10 is any of: { “F20.0”, “F20.1”, “F20.3”, “F20.81”, “F20.9”, “F21”, “F22”, “F24”, “F25.0”, “F25.8”, “F28”, “F95.2”, “F20.2”, “F20.5”, “F20.89”, “F23”, “F25.1”, “F25.9”, “F29”, “F95.8”, “F95.9” }
Bipolar disorder	Diagnosis Code, ICD-9 is any of: { “296.01”, “296.03”, “296.05”, “296.40”, “296.44”, “296.50”, “296.52”, “296.53”, “296.54”, “296.56”, “296.60”, “296.61”, “296.62”, “296.63”, “296.64”, “296.65”, “296.66”, “296.00”, “296.02”, “296.04”, “296.06”, “296.41”, “296.42”, “296.43”, “296.45”, “296.46”, “296.51”, “296.55”, “296.7”, “296.80”, “296.81”, “296.89” }<br>Diagnosis Code, ICD-10 is any of: { “F30.10”, “F30.3”, “F30.4”, “F30.8”, “F31.0”, “F31.10”, “F31.12”, “F31.30”, “F31.31”, “F31.60”, “F31.64”, “F31.71”, “F31.75”, “F31.77”, “F31.89”, “F30.11”, “F30.12”, “F30.13”, “F30.2”, “F30.9”, “F31.11”, “F31.13”, “F31.2”, “F31.32”, “F31.4”, “F31.5”, “F31.61”, “F31.62”, “F31.63”, “F31.70”, “F31.72”, “F31.73”, “F31.74”, “F31.76”, “F31.78”, “F31.81”, “F31.9” }
Depression	Diagnosis Code, ICD-9 is any of: { “296.20”, “296.21”, “296.22”, “296.25”, “296.32”, “296.33”, “296.34”, “296.36”, “296.90”, “298.0”, “296.23”, “296.24”, “296.30”, “296.31”, “296.35”, “296.82”, “296.99”, “311”, “296.26” }<br>Diagnosis Code, ICD-10 is any of: { “F32.4”, “F32.5”, “F32.89”, “F33.0”, “F33.1”, “F33.2”, “F33.3”, “F33.40”, “F33.41”, “F33.8”, “F34.89”, “F34.9”, “F32.0”, “F32.1”, “F32.2”, “F32.3”, “F32.9”, “F33.42”, “F33.9”, “F39” }

© 2023 Zhang Y et al. *JAMA Network Open*.

eTable 3. Eligible Infection Conditions for Cohort Inclusion

The definitions of the infections were drawn from published studies and verified by the board-certified physicians [JW and KJL]). The eligible types of infections included COVID-19,[1] influenza,[2] pneumonia[3,4], bacteremia[5], urinary tract infections (urinary tract infections,[6,7] prostatitis,[8,9] cystitis,[10-12] urethritis,[13] renal abscess,[14] pyelonephritis[15,16]), endocarditis,[17] soft tissue infection,[18,19] osteomyelitis,[20] septic arthritis,[21] central nervous system infection,[22,23] and intra-abdominal infections.[24]

**Infection Conditions**	**Codes/Definition**
COVID-19	Diagnosis Code, ICD-10 is any of: { "U07.1" }
Influenza	Diagnosis Code, ICD-9 is any of: { "487.1", "488", "488.01", "488.09", "488.1", "488.81", "488.89", "487", "487.0", "487.8", "488.0", "488.02", "488.11", "488.12", "488.19", "488.8", "488.82" }<br>Diagnosis Code, ICD-10 is any of: { "J09", "J09.X", "J10.00", "J10.01", "J10.08", "J10.1", "J10.2", "J10.83", "J11", "J11.0", "J11.2", "J11.82", "J11.83", "J11.89", "J09.X1", "J09.X2", "J09.X3", "J09.X9", "J10", "J10.0", "J10.8", "J10.81", "J10.82", "J10.89", "J11.00", "J11.08", "J11.1", "J11.81" }
UTI: urinary tract infections/prostatitis/cystitis/urethritis/renal abscess/pyelonephritis	Diagnosis Code (Any Confinement Position), ICD-9 is any of: { "590.00", "590.01", "590.11", "590.3", "590.80", "590.9", "590.10", "590.81", "599.0", "590.2", "601.1", "601.3", "601.4", "601.0", "601.8", "601.9", "595", "595.8", "098.11", "595.2", "595.3", "595.4", "595.89", "601.3", "595.0", "595.9", "599.0", "597.80", "597.89", "590.2", "590.0", "590.3", "590.80", "590.9", "590.1", "590.10", "590.2", "590.8", "590.81" }<br>Diagnosis Code, ICD-10 is any of: { "J09", "J09.X", "J10.00", "J10.01", "J10.08", "J10.1", "J10.2", "J10.83", "J11", "J11.0", "J11.2", "J11.82", "J11.83", "J11.89", "J09.X1", "J09.X2", "J09.X3", "J09.X9", "J10", "J10.0", "J10.8", "J10.81", "J10.82", "J10.89", "J11.00", "J11.08", "J11.1", "J11.81", "N41.2", "N41.4", "N41.8", "N41", "N41.0", "N41.3", "N41.9", "N30.0", "N30.01", "N30.2", "N30.21", "N30.8", "N30.80", "N30.81", "N30.9", "N30.91", "N30", "N30.00", "N30.20", "N30.90", "N39.0", "N34.1", "N34.2", "N39.0", "N15.1", "N10" }
Pneumonia	Diagnosis Code, ICD-9 is any of: { "010", "011", "031.0", "052.1", "466", "480.0", "480.1", "480.2", "480.8", "480.9", "481", "482.1", "482.2", "482.3", "482.31", "482.39", "482.41", "482.49", "482.81", "482.82", "482.84", "484.5", "484.6", "484.8", "488.01", "488.81", "507", "513", "006.4", "012", "055.1", "112.4", "480.3", "482.0", "482.32", "482.40", "482.42", "482.83", "482.89", "482.9", "483.0", "483.1", "483.8", "484.1", "484.3", "484.7", "485", "486", "487.0", "488.11", "510" }<br>Diagnosis Code, ICD-10 is any of: { "A06.5", "A15", "A37.11", "A42.0", "B25.0", "B38.0", "B38.1", "B38.2", "B39.2", "B46.0", "B77.81", "J10.00", "J10.01", "J10.08", "J11.0", "J12.1", "J12.8", "J12.81", "J12.89", "J13", "J15", "J15.0", "J15.2", "J15.3", "J15.5", "J15.9", "J16.0", "J17", "J18.0", "J18.2", "J18.8", "J18.9", "J22", "J69.0", "J69.1", "J69.8", "A22.1", "A37.01", "A37.81", "A37.91", "A43.0", "A48.1", "B01.2", "B05.2", "B37.1", "B39.0", "B39.1", "B40.0", "B40.1", "B40.2", "B44.0", "B59", "J09.X1", "J11.00", "J11.08", "J12", "J12.0", "J12.2", "J12.3", "J12.9", "J14", "J15.1", "J15.20", "J15.211", "J15.212", "J15.29", "J15.4", "J15.6", "J15.7", "J15.8", "J16", "J16.8", "J18", "J18.1", "J69", "J85", "J86" }

© 2023 Zhang Y et al. *JAMA Network Open*.

Bacteremia	Diagnosis Code, ICD-9 is any of: { “038.0”, “038.41”, “038.42”, “038.49”, “038.9”, “790.7”, “036.2”, “038.10”, “038.11”, “038.12”, “038.19”, “038.2”, “038.3”, “038.4”, “038.40”, “038.43”, “038.44”, “038.8” }<br>Diagnosis Code, ICD-10 is any of: { “A39.3”, “A39.4”, “I76”, “A39.2”, “R78.81” }
Endocarditis	Diagnosis Code, ICD-9 is any of: { “036.42”, “093.21”, “093.22”, “093.24”, “098.84”, “115.14”, “421.0”, “421.1”, “424.91”, “093.20”, “093.23”, “112.81”, “115.04”, “115.94”, “421.9”, “424.9”, “424.90”, “424.99” }<br>Diagnosis Code, ICD-10 is any of: { “A32.82”, “A39.51”, “A52.03”, “A54.83”, “I33.0”, “I38”, “B37.6”, “I33.9”, “I39” }
Soft tissue infection (decubitus/cellulitis)	Diagnosis Code, ICD-9 is any of: { “035”, “373.13”, “566”, “616.4”, “680.3”, “680.6”, “680.9”, “681.0”, “681.1”, “681.9”, “682.3”, “682.7”, “682.8”, “684”, “685.0”, “686.8”, “686.9”, “705.83”, “707.0”, “707.00”, “707.01”, “707.07”, “707.09”, “707.20”, “707.21”, “707.22”, “707.23”, “707.25”, “707.8”, “707.9”, “728.0”, “958.3”, “998.59”, “376.01”, “528.3”, “680”, “680.0”, “680.1”, “680.2”, “680.4”, “680.5”, “680.7”, “680.8”, “681”, “682”, “682.0”, “682.1”, “682.2”, “682.4”, “682.5”, “682.6”, “682.9”, “686”, “707”, “707.02”, “707.03”, “707.04”, “707.05”, “707.06”, “707.2”, “707.24”, “728.86”, “785.4”, “997.62”, “998.51” }<br>Diagnosis Code, ICD-10 is any of: { “A36.3”, “A43.1”, “A48.0”, “E09.52”, “E10.52”, “E11.52”, “E13.52”, “H00.031”, “H00.034”, “H00.035”, “H05.013”, “H05.019”, “H60.00”, “H60.11”, “H60.13”, “I70.261”, “I70.262”, “I70.263”, “I70.268”, “I70.269”, “I70.361”, “I70.368”, “I70.461”, “I70.468”, “I70.562”, “I70.563”, “I70.663”, “I70.668”, “I70.669”, “I70.763”, “I70.768”, “I96”, “J85.0”, “K12.2”, “K61.0”, “K61.1”, “K61.2”, “K61.4”, “L01.02”, “L02.02”, “L02.03”, “L02.12”, “L02.211”, “L02.212”, “L02.216”, “L02.219”, “L02.223”, “L02.224”, “L02.225”, “L02.226”, “L02.229”, “L02.231”, “L02.232”, “L02.236”, “L02.239”, “L02.31”, “L02.32”, “L02.414”, “L02.415”, “L02.416”, “L02.419”, “L02.425”, “L02.426”, “L02.429”, “L02.431”, “L02.433”, “L02.434”, “L02.436”, “L02.439”, “L02.519”, “L02.521”, “L02.522”, “L02.529”, “L02.531”, “L02.532”, “L02.539”, “L02.612”, “L02.619”, “L02.621”, “L02.632”, “L02.639”, “L02.831”, “L02.91”, “L02.92”, “L02.93”, “L03.011”, “L03.012”, “L03.031”, “L03.032”, “L03.114”, “L03.115”, “L03.213”, “L03.311”, “L03.312”, “L03.314”, “L03.315”, “L05.92”, “L08”, “L08.9”, “L89.00”, “L89.01”, “L89.10”, “L89.14”, “L89.20”, “L89.21”, “L89.22”, “L89.30”, “L89.31”, “L89.32”, “L89.40”, “L89.43”, “L89.46”, “L89.52”, “L89.60”, “L89.81”, “L89.90”, “L89.93”, “L89.95”, “L89.96”, “L98.3”, “N48.22”, “N73.2”, “A18.01”, “A31.1”, “A46”, “E08.52”, “H00.032”, “H00.033”, “H00.036”, “H00.039”, “H05.011”, “H05.012”, “H60.01”, “H60.02”, “H60.03”, “H60.10”, “H60.12”, “I70.362”, “I70.363”, “I70.369”, “I70.462”, “I70.463”, “I70.469”, “I70.561”, “I70.568”, “I70.569”, “I70.661”, “I70.662”, “I70.761”, “I70.762”, “I70.769”, “I73.01”, “J34.0”, “K61.3”, “L01.00”, “L01.01”, “L01.03”, “L01.09”, “L01.1”, “L02.01”, “L02.11”, “L02.13”, “L02.213”, “L02.214”, “L02.215”, “L02.221”, “L02.222”, “L02.233”, “L02.234”, “L02.235”, “L02.33”, “L02.411”, “L02.412”, “L02.413”, “L02.421”, “L02.422”, “L02.423”, “L02.424”, “L02.432”, “L02.435”, “L02.511”, “L02.512”, “L02.611”, “L02.622”, “L02.629”, “L02.631”, “L02.811”, “L02.818”, “L02.821”, “L02.828”, “L02.838”, “L03.0”, “L03.019”, “L03.039”, “L03.1”, “L03.111”, “L03.112”, “L03.113”, “L03.116”, “L03.119”, “L03.211”, “L03.221”, “L03.313”, “L03.316”, “L03.317”, “L03.319”, “L03.811”, “L03.818”, “L03.90”, “L05.01”, “L05.02”, “L05.91”, “L08.89”, “L89.02”, “L89.11”, “L89.12”, “L89.13”, “L89.15”, “L89.41”, “L89.42”, “L89.44”, “L89.45”, “L89.50”, “L89.51”, “L89.61”, “L89.62”, “L89.89”, “L89.91”, “L89.92”, “L89.94”, “M72.6”, “N48.21”, “N61.1”, “N73.0” }

© 2023 Zhang Y et al. *JAMA Network Open*.

Osteomyelitis/septic arthritis	Diagnosis Code, ICD-9 is any of: { “003.24”, “711.03”, “711.07”, “730.00”, “730.01”, “730.05”, “730.07”, “730.10”, “730.13”, “730.18”, “730.20”, “730.21”, “730.23”, “730.24”, “730.25”, “730.29”, “730.80”, “730.81”, “730.83”, “730.84”, “730.85”, “730.87”, “730.89”, “376.03”, “711.0”, “711.00”, “711.01”, “711.02”, “711.04”, “711.05”, “711.06”, “711.08”, “711.09”, “730.02”, “730.03”, “730.04”, “730.06”, “730.08”, “730.09”, “730.11”, “730.12”, “730.14”, “730.15”, “730.16”, “730.17”, “730.19”, “730.22”, “730.26”, “730.27”, “730.28”, “730.82”, “730.86”, “730.88” }<br>Diagnosis Code, ICD-10 is any of: { “M00”, “M00.0”, “M00.00”, “M00.011”, “M00.012”, “M00.029”, “M00.04”, “M00.042”, “M00.049”, “M00.051”, “M00.06”, “M00.061”, “M00.062”, “M00.072”, “M00.079”, “M00.1”, “M00.10”, “M00.111”, “M00.12”, “M00.13”, “M00.132”, “M00.14”, “M00.141”, “M00.142”, “M00.152”, “M00.159”, “M00.162”, “M00.169”, “M00.18”, “M00.2”, “M00.211”, “M00.212”, “M00.219”, “M00.221”, “M00.222”, “M00.229”, “M00.232”, “M00.242”, “M00.25”, “M00.26”, “M00.261”, “M00.27”, “M00.271”, “M00.28”, “M00.8”, “M00.811”, “M00.812”, “M00.819”, “M00.821”, “M00.822”, “M00.83”, “M00.831”, “M00.832”, “M00.839”, “M00.84”, “M00.842”, “M00.85”, “M00.859”, “M00.86”, “M00.87”, “M00.871”, “M00.872”, “M00.88”, “M00.01”, “M00.019”, “M00.02”, “M00.021”, “M00.022”, “M00.03”, “M00.031”, “M00.032”, “M00.039”, “M00.041”, “M00.05”, “M00.052”, “M00.059”, “M00.069”, “M00.07”, “M00.071”, “M00.08”, “M00.09”, “M00.11”, “M00.112”, “M00.119”, “M00.121”, “M00.122”, “M00.129”, “M00.131”, “M00.139”, “M00.149”, “M00.15”, “M00.151”, “M00.16”, “M00.161”, “M00.17”, “M00.171”, “M00.172”, “M00.179”, “M00.19”, “M00.20”, “M00.21”, “M00.22”, “M00.23”, “M00.231”, “M00.239”, “M00.24”, “M00.241”, “M00.249”, “M00.251”, “M00.252”, “M00.259”, “M00.262”, “M00.269”, “M00.272”, “M00.279”, “M00.29”, “M00.80”, “M00.81”, “M00.82”, “M00.829”, “M00.841”, “M00.849”, “M00.851”, “M00.852”, “M00.861”, “M00.862”, “M00.869”, “M00.879”, “M00.89”, “M00.9”, “M01”, “M01.X12”, “M01.X19”, “M01.X21”, “M01.X3”, “M01.X31”, “M01.X39”, “M01.X41”, “M01.X42”, “M01.X49”, “M01.X51”, “M01.X59”, “M01.X69”, “M01.X79”, “M01.X8”, “M01.X”, “M01.X0”, “M01.X1”, “M01.X11”, “M01.X2”, “M01.X22”, “M01.X29”, “M01.X32”, “M01.X4”, “M01.X5”, “M01.X52”, “M01.X6”, “M01.X61”, “M01.X62”, “M01.X7”, “M01.X71”, “M01.X72”, “M01.X9”, “M02”, “M02.0”, “M02.00”, “M02.01”, “M02.019”, “M02.022”, “M02.03”, “M02.032”, “M02.039”, “M02.04”, “M02.041”, “M02.05”, “M02.051”, “M02.052”, “M02.059”, “M02.06”, “M02.062”, “M02.07”, “M02.071”, “M02.072”, “M02.08”, “M02.1”, “M02.10”, “M02.11”, “M02.121”, “M02.122”, “M02.129”, “M02.13”, “M02.131”, “M02.139”, “M02.14”, “M02.141”, “M02.149”, “M02.151”, “M02.159”, “M02.16”, “M02.169”, “M02.17”, “M02.171”, “M02.179”, “M02.2”, “M02.211”, “M02.212”, “M02.219”, “M02.221”, “M02.222”, “M02.23”, “M02.242”, “M02.252”, “M02.262”, “M02.272”, “M02.279”, “M02.29”, “M02.3”, “M02.30”, “M02.31”, “M02.311”, “M02.312”, “M02.319”, “M02.321”, “M02.322”, “M02.329”, “M02.33”, “M02.332”, “M02.339”, “M02.34”, “M02.341”, “M02.342”, “M02.36”, “M02.362”, “M02.369”, “M02.37”, “M02.371”, “M02.372”, “M02.379”, “M02.39”, “M02.8”, “M02.81”, “M02.811”, “M02.82”, “M02.821”, “M02.83”, “M02.831”, “M02.832”, “M02.839”, “M02.84”, “M02.841”, “M02.842”, “M02.849”, “M02.851”, “M02.852”, “M02.859”, “M02.86”, “M02.869”, “M02.87”, “M02.872”, “M02.9”, “M02.011”, “M02.012”, “M02.02”, “M02.021”, “M02.029”, “M02.031”, “M02.042”, “M02.049”, “M02.061”, “M02.069”, “M02.079”, “M02.09”, “M02.111”, “M02.112”, “M02.119”, “M02.12”, “M02.132”, “M02.142”, “M02.15”, “M02.152”, “M02.161”, “M02.162”, “M02.172”, “M02.18”, “M02.19”, “M02.20”, “M02.21”, “M02.22”, “M02.229”, “M02.231”, “M02.232”, “M02.239”,

© 2023 Zhang Y et al. *JAMA Network Open*.

	"M02.24", "M02.241", "M02.249", "M02.25", "M02.251", "M02.259", "M02.26", "M02.261", "M02.269", "M02.27", "M02.271", "M02.28", "M02.32", "M02.331", "M02.349", "M02.35", "M02.351", "M02.352", "M02.359", "M02.361", "M02.38", "M02.80", "M02.812", "M02.819", "M02.822", "M02.829", "M02.85", "M02.861", "M02.862", "M02.871", "M02.879", "M02.88", "M02.89", "H05.022", "H05.023", "M46.20", "M46.22", "M46.25", "M46.28", "M86.00", "M86.012", "M86.021", "M86.031", "M86.032", "M86.041", "M86.051", "M86.072", "M86.08", "M86.09", "M86.119", "M86.121", "M86.122", "M86.129", "M86.142", "M86.162", "M86.169", "M86.171", "M86.172", "M86.179", "M86.20", "M86.211", "M86.212", "M86.229", "M86.232", "M86.241", "M86.242", "M86.251", "M86.252", "M86.259", "M86.261", "M86.262", "M86.269", "M86.271", "M86.272", "M86.28", "M86.30", "M86.319", "M86.331", "M86.339", "M86.342", "M86.352", "M86.359", "M86.361", "M86.369", "M86.372", "M86.379", "M86.40", "M86.429", "M86.431", "M86.432", "M86.439", "M86.441", "M86.442", "M86.459", "M86.469", "M86.472", "M86.479", "M86.519", "M86.522", "M86.532", "M86.539", "M86.551", "M86.559", "M86.562", "M86.569", "M86.59", "M86.60", "M86.611", "M86.621", "M86.622", "M86.631", "M86.639", "M86.642", "M86.649", "M86.652", "M86.659", "M86.661", "M86.662", "M86.679", "M86.8X0", "M86.8X3", "M86.8X5", "A01.05", "A02.24", "H05.021", "H05.029", "M46.21", "M46.23", "M46.24", "M46.26", "M46.27", "M72.6", "M86.011", "M86.019", "M86.022", "M86.029", "M86.039", "M86.042", "M86.049", "M86.052", "M86.059", "M86.061", "M86.062", "M86.069", "M86.071", "M86.079", "M86.10", "M86.111", "M86.112", "M86.131", "M86.132", "M86.139", "M86.141", "M86.149", "M86.151", "M86.152", "M86.159", "M86.161", "M86.18", "M86.19", "M86.219", "M86.221", "M86.222", "M86.231", "M86.239", "M86.249", "M86.279", "M86.29", "M86.311", "M86.312", "M86.321", "M86.322", "M86.329", "M86.332", "M86.341", "M86.349", "M86.351", "M86.362", "M86.371", "M86.38", "M86.39", "M86.411", "M86.412", "M86.419", "M86.421", "M86.422", "M86.449", "M86.451", "M86.452", "M86.461", "M86.462", "M86.471", "M86.48", "M86.49", "M86.50", "M86.511", "M86.512", "M86.521", "M86.529", "M86.531", "M86.541", "M86.542", "M86.549", "M86.552", "M86.561", "M86.571", "M86.572", "M86.579", "M86.58", "M86.612", "M86.619", "M86.629", "M86.632", "M86.641", "M86.651", "M86.669", "M86.671", "M86.672", "M86.68", "M86.69", "M86.8X1", "M86.8X2", "M86.8X4", "M86.8X6", "M86.8X7", "M86.8X8", "M86.8X9", "M86.9" }
CNS infection (meningitis, encephalitis, epidural abscess, brain abscess)	Diagnosis Code, ICD-9 is any of: { "003.21", "006.5", "013", "036.0", "036.1", "045.02", "045.1", "045.10", "045.11", "045.20", "045.21", "045.23", "045.9", "045.91", "045.92", "045.93", "046", "046.19", "046.2", "046.3", "046.71", "046.8", "047", "047.0", "047.1", "047.9", "048", "049.1", "052.0", "053.0", "053.1", "054.3", "063", "064", "072.2", "091.81", "320.0", "320.1", "320.2", "320.7", "320.89", "320.9", "036.2", "045", "045.0", "045.00", "045.01", "045.03", "045.12", "045.13", "045.2", "045.22", "045.90", "046.0", "046.1", "046.11", "046.7", "046.72", "046.79", "046.9", "047.8", "049", "049.0", "049.8", "049.9", "054.7", "055.0", "056.0", "062", "072.1", "094.2", "094.81", "098.82", "320.3", "320.81", "320.82", "323.1", "323.2", "323.9" }<br>Diagnosis Code, ICD-10 is any of: { "A17", "A39", "A39.0", "A52.13", "A52.14", "A80.0", "A80.2", "A80.30", "A80.9", "A81.00", "A81.01", "A81.1", "A81.8", "A81.83", "A81.89", "A81.9", "A82", "A82.0", "A82.1", "A82.9", "A83.0", "A84", "A84.0", "A84.8", "A84.89", "A85.0", "A85.8", "A87", "A87.8", "A88.8", "B00.3", "B01.1", "B05.1", "B06.0", "B26.1", "B26.2", "B38.4", "B58.2", "G00", "G00.3", "G00.9", "G01", "G02.0", "G03", "G03.0", "G04", "G04.8", "G04.9", "G06", "G07", "A02.21", "A06.6", "A39.81", "A51.41", "A54.81", "A80", "A80.1", "A80.3", "A80.39", "A80.4", "A81", "A81.0", "A81.09", "A81.2", "A81.81", "A81.82",

© 2023 Zhang Y et al. *JAMA Network Open*.

	"A83", "A84.1", "A84.81", "A84.9", "A85", "A85.2", "A86", "A87.0", "A87.2", "A87.9", "A88", "A88.0", "A88.1", "A89", "B00.4", "B01.0", "B02.0", "B02.1", "B05.0", "B37.5", "B43.1", "B50.0", "B60.2", "G00.0", "G00.1", "G00.2", "G00.8", "G02", "G03.9", "G04.0", "G04.2", "G05" }
Intra-abdominal infections	Diagnosis Code, ICD-9 is any of: { "531.10", "531.20", "531.21", "531.51", "531.60", "531.61", "532.10", "532.20", "532.50", "532.51", "532.60", "532.61", "533.50", "533.51", "534.10", "534.20", "534.21", "534.60", "540.1", "567.0", "567.3", "567.8", "567.9", "569.81", "572", "574.8", "531.11", "531.50", "532.11", "532.21", "533.10", "533.11", "533.20", "533.21", "533.60", "533.61", "534.11", "534.50", "534.51", "534.61", "540", "567.1", "567.2", "569.5", "569.82", "569.83", "574.0", "574.3", "574.6", "575.0", "575.4" }<br>Diagnosis Code, ICD-10 is any of: { "K25.1", "K26.1", "K26.2", "K26.5", "K27.1", "K28.2", "K28.6", "K35.3", "K63.1", "K65.0", "K65.1", "K65.8", "K80.0", "K80.00", "K80.01", "K80.1", "K80.12", "K80.13", "K80.3", "K80.30", "K80.35", "K80.36", "K80.4", "K80.41", "K80.42", "K80.43", "K80.47", "K80.6", "K80.63", "K80.66", "K80.67", "K80.81", "K25.2", "K25.5", "K25.6", "K26.6", "K27.2", "K27.5", "K27.6", "K28.1", "K28.5", "K35.2", "K63.0", "K65.3", "K67", "K75.0", "K80.18", "K80.19", "K80.31", "K80.32", "K80.33", "K80.34", "K80.37", "K80.40", "K80.46", "K80.60", "K80.61", "K80.62" }

© 2023 Zhang Y et al. *JAMA Network Open*.

eTable 4. List of Covariates

The definitions of the covariates were drawn from published studies and verified by the board-certified physicians [JW and KJL]). In the 180 days before cohort entry, we assessed baseline covariates, including demographic factors, baseline conditions,[25] healthcare utilization,[25] frailty measured using a claims-based frailty index (CFI) validated against clinical measures of frailty[26], and calendar year.

**Covariates**	**Codes/Definition**
**Frailty Score**	Claims-based frailty index according to Kim DH, Schneeweiss S, Lipsitz LA, Glynn R, Rockwood K, Avorn J. Measuring Frailty in Medicare Data: Development and Validation of a Claims-Based Frailty Index. *J Gerontol A Biol Sci Med Sci.* 2018; 73: 980-987. doi: 10.1093/gerona/glx229.
**Comorbidities**	
**Dementia**	Diagnosis Code, ICD-9 is any of: { "290.1", "290.11", "290.20", "290.21", "290.42", "290.43", "294.10", "294.8", "331.19", "290.0", "290.12", "290.13", "290.3", "290.40", "290.41", "294.0", "294.11", "294.20", "294.21", "331.0", "331.11", "331.2", "331.7", "797" }<br>Diagnosis Code ICD-10 is any of: { "F01.50", "F02.8", "F03.90", "G30.8", "G30.9", "G31.0", "G31.09", "F01.5", "F01.51", "F02.80", "F02.81", "F03.9", "F03.91", "G30.0", "G30.1", "G31.01" }
**Heart failure**	Diagnosis Code, ICD-9 is any of: { “398.91”, “402.01”, “402.91”, “404.03”, “404.11”, “404.13”, “428.0”, “428.1”, “428.2”, “428.3”, “428.41”, “428.42”, “428.43”, “428.9”, “402.11”, “404.01”, “404.93”, “428”, “428.20”, “428.21”, “428.22”, “428.23”, “428.30”, “428.31”, “428.32”, “428.33”, “428.4”, “428.40” }<br>Diagnosis Code, ICD-10 is any of: { “I09.81”, “I11.0”, “I13.0”, “I13.2”, “I50.1”, “I50.21”, “I50.22”, “I50.23”, “I50.30”, “I50.31”, “I50.40”, “I50.42”, “I50.43”, “I50.20”, “I50.32”, “I50.33”, “I50.41”, “I50.9” }
**Hypertension**	Diagnosis Code, ICD-9 is any of: { “401.0”, “401.1”, “401.9”, “402.0”, “402.00”, “402.01”, “402.10”, “402.90”, “402.91”, “403”, “403.00”, “403.11”, “403.9”, “403.91”, “404”, “404.02”, “404.03”, “404.1”, “404.10”, “404.11”, “404.13”, “404.9”, “404.91”, “404.92”, “405.19”, “401”, “402”, “402.1”, “402.11”, “402.9”, “403.0”, “403.01”, “403.1”, “403.10”, “403.90”, “404.0”, “404.00”, “404.01”, “404.12”, “404.90”, “404.93”, “405”, “405.0”, “405.01”, “405.09”, “405.1”, “405.11”, “405.9”, “405.91”, “405.99” }<br>Diagnosis Code, ICD-10 is any of: { “I11.0”, “I13.0”, “I13.10”, “I13.2”, “I15.0”, “I15.2”, “I15.8”, “I15.9”, “N26.2”, “I10”, “I11.9”, “I12.0”, “I12.9”, “I13.11”, “I15.1” }
**Ischemic heart disease**	Diagnosis Code, ICD-9 is any of: { “410.0”, “410.01”, “410.1”, “410.11”, “410.20”, “410.22”, “410.3”, “410.31”, “410.40”, “410.42”, “410.51”, “410.52”, “410.6”, “410.60”, “410.71”, “410.72”, “410.80”, “410.81”, “410.82”, “410.9”, “410.90”, “410.91”, “411.0”, “411.1”, “411.8”, “411.89”, “413.1”, “414.0”, “414.05”, “414.06”, “414.10”, “414.11”, “414.12”, “414.2”, “414.4”, “410.00”, “410.02”, “410.10”, “410.12”, “410.2”, “410.21”, “410.30”, “410.32”, “410.4”, “410.41”, “410.5”, “410.50”, “410.61”, “410.62”, “410.7”, “410.70”, “410.8”, “410.92”, “411.81”, “412”, “413.0”, “413.9”, “414.00”, “414.01”, “414.02”, “414.03”, “414.04”, “414.07”, “414.1”, “414.19”, “414.3”, “414.8”, “414.9” }

© 2023 Zhang Y et al. *JAMA Network Open*.

	Diagnosis Code, ICD-10 is any of: { “I20.0”, “I20.1”, “I20.8”, “I22.0”, “I22.1”, “I22.2”, “I22.8”, “I24.0”, “I24.9”, “I25.10”, “I25.110”, “I25.111”, “I25.118”, “I25.2”, “I25.41”, “I25.42”, “I25.5”, “I25.709”, “I25.710”, “I25.711”, “I25.718”, “I25.719”, “I25.721”, “I25.729”, “I25.730”, “I25.738”, “I25.759”, “I25.768”, “I25.769”, “I25.790”, “I25.791”, “I25.83”, “I20.9”, “I21.01”, “I21.02”, “I21.09”, “I21.11”, “I21.19”, “I21.21”, “I21.29”, “I21.3”, “I21.4”, “I22.9”, “I24.1”, “I24.8”, “I25.119”, “I25.3”, “I25.6”, “I25.700”, “I25.701”, “I25.708”, “I25.720”, “I25.728”, “I25.731”, “I25.739”, “I25.750”, “I25.751”, “I25.758”, “I25.760”, “I25.761”, “I25.798”, “I25.799”, “I25.810”, “I25.811”, “I25.812”, “I25.82”, “I25.84”, “I25.89”, “I25.9” }
**Falls**	Diagnosis Code, ICD-9 is any of: { “E880”, “E880.1”, “E880.9”, “E881.0”, “E881.1”, “E882”, “E883”, “E883.2”, “E883.9”, “E884”, “E884.2”, “E884.3”, “E884.5”, “E885.1”, “E885.2”, “E885.3”, “E885.9”, “E887”, “E888.0”, “E888.1”, “E888.8”, “E880.0”, “E881”, “E883.0”, “E883.1”, “E884.0”, “E884.1”, “E884.4”, “E884.6”, “E884.9”, “E885”, “E885.0”, “E885.4”, “E886”, “E886.0”, “E886.9”, “E888”, “E888.9” }<br>Diagnosis Code ICD-10 is any of: { “V00.111D”, “V00.112A”, “V00.112D”, “V00.118A”, “V00.118D”, “V00.121A”, “V00.121D”, “V00.122D”, “V00.128A”, “V00.131A”, “V00.131D”, “V00.132A”, “V00.138D”, “V00.141D”, “V00.148A”, “V00.148D”, “V00.152A”, “V00.152D”, “V00.181A”, “V00.181D”, “V00.182D”, “V00.188A”, “V00.211A”, “V00.212A”, “V00.218D”, “V00.221A”, “V00.222D”, “V00.228D”, “V00.281A”, “V00.282D”, “V00.288A”, “V00.318D”, “V00.321A”, “V00.321D”, “V00.322A”, “V00.322D”, “V00.328A”, “V00.381D”, “V00.388A”, “V00.811D”, “V00.812D”, “V00.818D”, “V00.822A”, “V00.828A”, “V00.828D”, “V00.832D”, “V00.838A”, “V00.891A”, “V00.891D”, “V00.892A”, “V00.898D”, “W00.1XXA”, “W01.0XXA”, “W01.10XA”, “W01.10XD”, “W01.118A”, “W01.118D”, “W01.198D”, “W04.XXXA”, “W04.XXXD”, “W05.1XXA”, “W05.1XXD”, “W05.2XXA”, “W05.2XXD”, “W06.XXXA”, “W06.XXXD”, “W07.XXXD”, “W09.0XXA”, “W09.0XXD”, “W09.1XXA”, “W09.1XXD”, “W09.2XXD”, “W09.8XXA”, “W09.8XXD”, “W10.0XXA”, “W10.2XXD”, “W10.8XXA”, “W10.9XXA”, “W10.9XXD”, “W12.XXXD”, “W13.0XXA”, “W13.2XXA”, “W13.3XXD”, “W13.4XXD”, “W13.8XXA”, “W13.8XXD”, “W13.9XXA”, “W13.9XXD”, “W14.XXXD”, “W16.011A”, “W16.011D”, “W16.012A”, “W16.021A”, “W16.021D”, “W16.022A”, “W16.022D”, “W16.031A”, “W16.032D”, “W16.111A”, “W16.111D”, “W16.132A”, “W16.211A”, “W16.211D”, “W16.212D”, “W16.221D”, “W16.311A”, “W16.311D”, “W16.322A”, “W16.41XD”, “W16.42XA”, “W16.42XD”, “W16.511A”, “W16.511D”, “W16.512D”, “W16.521A”, “W16.521D”, “W16.531D”, “W16.532A”, “W16.532D”, “W16.611D”, “W16.621A”, “W16.621D”, “W16.622A”, “W16.721D”, “W16.811A”, “W16.812A”, “W16.821D”, “W16.822A”, “W16.822D”, “W16.831A”, “W16.831D”, “W16.832A”, “W16.832D”, “W16.92XA”, “W16.92XD”, “W17.0XXA”, “W17.0XXD”, “W17.1XXD”, “W17.3XXA”, “W17.4XXA”, “W17.4XXD”, “W17.81XA”, “W17.82XA”, “W17.82XD”, “W17.89XA”, “W17.89XD”, “W18.00XA”, “W18.01XD”, “W18.02XA”, “W18.02XD”, “W18.12XD”, “W18.2XXD”, “W18.30XA”, “W18.30XD”, “W18.40XD”, “W18.41XA”, “W18.42XA”, “W18.42XD”, “W18.43XA”, “W18.43XD”, “W18.49XA”, “W18.49XD”, “W19.XXXA”, “V00.111A”, “V00.122A”, “V00.128D”, “V00.132D”, “V00.138A”, “V00.141A”, “V00.142A”, “V00.142D”, “V00.151A”, “V00.151D”, “V00.158A”, “V00.158D”, “V00.182A”, “V00.188D”, “V00.211D”, “V00.212D”, “V00.218A”, “V00.221D”, “V00.222A”, “V00.228A”, “V00.281D”, “V00.282A”, “V00.288D”, “V00.311A”, “V00.311D”, “V00.312A”, “V00.312D”, “V00.318A”, “V00.328D”, “V00.381A”, “V00.382A”,

© 2023 Zhang Y et al. *JAMA Network Open*.

	"V00.382D", "V00.388D", "V00.811A", "V00.812A", "V00.818A", "V00.821A", "V00.821D", "V00.822D", "V00.831A", "V00.831D", "V00.832A", "V00.838D", "V00.892D", "V00.898A", "V15.88", "W00.0XXA", "W00.0XXD", "W00.1XXD", "W00.2XXA", "W00.2XXD", "W00.9XXA", "W00.9XXD", "W01.0XXD", "W01.110A", "W01.110D", "W01.111A", "W01.111D", "W01.119A", "W01.119D", "W01.190A", "W01.190D", "W01.198A", "W03.XXXA", "W03.XXXD", "W05.0XXA", "W05.0XXD", "W07.XXXA", "W08.XXXA", "W08.XXXD", "W09.2XXA", "W10.0XXD", "W10.1XXA", "W10.1XXD", "W10.2XXA", "W10.8XXD", "W11.XXXA", "W11.XXXD", "W12.XXXA", "W13.0XXD", "W13.1XXA", "W13.1XXD", "W13.2XXD", "W13.3XXA", "W13.4XXA", "W14.XXXA", "W15.XXXA", "W15.XXXD", "W16.012D", "W16.031D", "W16.032A", "W16.112A", "W16.112D", "W16.121A", "W16.121D", "W16.122A", "W16.122D", "W16.131A", "W16.131D", "W16.132D", "W16.212A", "W16.221A", "W16.222A", "W16.222D", "W16.312A", "W16.312D", "W16.321A", "W16.321D", "W16.322D", "W16.331A", "W16.331D", "W16.332A", "W16.332D", "W16.41XA", "W16.512A", "W16.522A", "W16.522D", "W16.531A", "W16.611A", "W16.612A", "W16.612D", "W16.622D", "W16.711A", "W16.711D", "W16.712A", "W16.712D", "W16.721A", "W16.722A", "W16.722D", "W16.811D", "W16.812D", "W16.821A", "W16.91XA", "W16.91XD", "W17.1XXA", "W17.2XXA", "W17.2XXD", "W17.3XXD", "W17.81XD", "W18.00XD", "W18.01XA", "W18.09XD", "W18.11XA", "W18.11XD", "W18.12XA", "W18.2XXA", "W18.31XA", "W18.31XD", "W18.39XA", "W18.39XD", "W18.40XA", "W18.41XD", "W19.XXXD", "Z91.81" }
**CKD**	Diagnosis Code, ICD-9 is any of: { "582", "582.0", "582.2", "582.4", "582.81", "582.89", "582.9", "583", "583.1", "583.6", "583.9", "585", "585.2", "585.3", "585.4", "585.5", "582.1", "582.8", "583.0", "583.2", "583.4", "583.7", "583.8", "583.81", "583.89", "585.1", "585.6", "585.9", "586", "587" }<br>Diagnosis Code, ICD-10 is any of: { "M32.15", "M35.04", "N03.0", "N03.2", "N03.3", "N03.5", "N03.7", "N03.9", "N05.0", "N05.2", "N05.5", "N05.6", "N05.8", "N06.2", "N06.4", "N06.5", "N06.7", "N06.8", "N07.0", "N07.1", "N07.4", "N07.6", "N07.7", "N07.9", "N08", "N14.0", "N14.2", "N14.3", "N15.0", "N15.8", "N17.1", "N18.5", "N18.9", "N19", "N26.9", "M32.14", "N03.1", "N03.4", "N03.6", "N03.8", "N05.1", "N05.3", "N05.4", "N05.7", "N05.9", "N06.0", "N06.1", "N06.3", "N06.6", "N06.9", "N07.2", "N07.3", "N07.5", "N07.8", "N14.1", "N14.4", "N15.9", "N16", "N17.2", "N18.1", "N18.2", "N18.3", "N18.4", "N18.6", "N26.1", "E09.21", "E09.22", "E09.29" }
**Cancer**	Diagnosis Code, ICD-9 is any of: { "233.2", "V10.42", "182.0", "153.0", "153.5", "153.6", "153.8", "153.9", "154.0", "154.1", "230.4", "V10.06", "153.1", "153.2", "153.3", "153.4", "153.7", "230.3", "V10.05", "162.4", "162.8", "V10.11", "162.2", "162.3", "162.5", "162.9", "231.2", "174.1", "174.5", "174.9", "175.9", "233.0", "V10.3", "174.0", "174.2", "174.3", "174.4", "174.6", "174.8", "175.0", "185", "233.4", "V10.46" }<br>Diagnosis Code, ICD-10 is any of: { "C54.2", "C54.3", "C54.8", "Z85.42", "C54.1", "C54.9", "D07.0", "C18.1", "C18.3", "C18.5", "C19", "C20", "D01.0", "Z85.038", "Z85.040", "Z85.048", "C18.0", "C18.2", "C18.4", "C18.6", "C18.7", "C18.8", "C18.9", "D01.1", "D01.2", "C34.02", "C34.10", "C34.11", "C34.12", "C34.2", "C34.30", "C34.31", "D02.20", "D02.21", "C34.00", "C34.01", "C34.32", "C34.80", "C34.81", "C34.82", "C34.90", "C34.91", "C34.92", "D02.22", "Z85.110", "Z85.118", "C50.011", "C50.012", "C50.119", "C50.121", "C50.211",

© 2023 Zhang Y et al. *JAMA Network Open*.

	"C50.219", "C50.222", "C50.311", "C50.312", "C50.321", "C50.322", "C50.329", "C50.411", "C50.412", "C50.419", "C50.429", "C50.512", "C50.521", "C50.522", "C50.529", "C50.612", "C50.622", "C50.629", "C50.811", "C50.812", "C50.819", "C50.821", "C50.822", "C50.911", "C50.912", "C50.919", "C50.921", "C50.929", "D05.02", "D05.11", "D05.12", "D05.80", "D05.82", "D05.90", "Z85.3", "C50.019", "C50.021", "C50.022", "C50.029", "C50.111", "C50.112", "C50.122", "C50.129", "C50.212", "C50.221", "C50.229", "C50.319", "C50.421", "C50.422", "C50.511", "C50.519", "C50.611", "C50.619", "C50.621", "C50.829", "C50.922", "D05.00", "D05.01", "D05.10", "D05.81", "D05.91", "D05.92", "D07.5", "C61", "Z85.46" }
**DVT**	Diagnosis Code, ICD-9 is any of: { "415.1", "451.81", "453.1", "453.84", "453.86", "453.87", "453.9", "451.11", "451.2", "451.9", "453.2", "453.8", "453.82", "453.83", "453.85", "453.89" }<br>Diagnosis Code, ICD-10 is any of: { "I80.219", "I82.1", "I82.221", "I82.609", "I82.629", "I82.91", "I82.B19", "I80.10", "I80.3", "I80.9", "I82.220", "I82.290", "I82.890", "I82.A19", "I82.C19" }
**Anemia**	Diagnosis Code (Any Confinement Position), ICD-9 is any of: { "280.9", "281.3", "281.4", "281.9", "282.1", "282.2", "282.3", "282.42", "282.44", "282.45", "282.49", "282.60", "282.63", "282.8", "282.9", "283.0", "283.2", "283.9", "284.09", "284.11", "284.2", "284.9", "285.0", "285.3", "285.8", "280.0", "280.1", "280.8", "281.0", "281.1", "281.2", "281.8", "282.0", "282.40", "282.41", "282.43", "282.46", "282.47", "282.5", "282.61", "282.62", "282.64", "282.68", "282.69", "282.7", "283.10", "283.11", "283.19", "284.01", "284.12", "284.19", "284.81", "284.89", "285.1", "285.21", "285.22", "285.29", "285.9" }<br>Diagnosis Code, ICD-10 is any of: { "D50.0", "D51.1", "D51.2", "D51.9", "D52.8", "D52.9", "D53.0", "D53.2", "D53.8", "D55.0", "D55.1", "D55.2", "D56.0", "D56.1", "D56.3", "D56.4", "D56.5", "D57.00", "D57.01", "D57.1", "D57.212", "D57.3", "D57.40", "D57.411", "D57.80", "D57.819", "D58.0", "D58.2", "D59.3", "D60.9", "D61.01", "D61.09", "D61.2", "D61.82", "D61.89", "D61.9", "D63.0", "D63.8", "D64.1", "D64.2", "D64.4", "D64.81", "D64.89", "D64.9", "D50.1", "D50.8", "D50.9", "D51.0", "D51.3", "D51.8", "D52.0", "D52.1", "D53.1", "D53.9", "D55.3", "D55.8", "D55.9", "D56.2", "D56.8", "D56.9", "D57.02", "D57.20", "D57.211", "D57.219", "D57.412", "D57.419", "D57.811", "D57.812", "D58.1", "D58.8", "D58.9", "D59.0", "D59.1", "D59.2", "D59.4", "D59.5", "D59.6", "D59.8", "D59.9", "D60.0", "D60.1", "D60.8", "D61.1", "D61.3", "D61.810", "D61.811", "D61.818", "D62", "D63.1", "D64.0", "D64.3" }
**Atrial fibrillation**	Diagnosis Code, ICD-9 is any of: { "427.32", "427.3", "427.31" }<br>Diagnosis Code, ICD-10 is any of: { "I48.0", "I48.1", "I48.4", "I48.92", "I48.2", "I48.3", "I48.91" }
**Liver Disease**	Diagnosis Code, ICD-9 is any of: { "070.2", "070.20", "070.22", "070.33", "070.4", "070.42", "070.51", "070.52", "070.53", "070.54", "070.9", "456.2", "571.1", "571.2", "571.40", "571.42", "571.49", "571.5", "572.4", "572.8", "573.0", "573.9", "576.8", "782.4", "789.59", "070.0", "070.1", "070.21", "070.23", "070.3", "070.30", "070.31", "070.32", "070.41", "070.43", "070.44", "070.49", "070.5", "070.59", "070.6", "070.7", "070.70", "070.71", "456.0", "456.1", "456.20", "456.21", "570", "571.0", "571.3", "571.4", "571.41", "571.6", "571.8", "571.9", "572.0", "572.1", "572.2", "572.3", "573.1", "573.2", "573.3", "573.4", "573.5", "573.8", "789.5", "789.51" }<br>Diagnosis Code, ICD-10 is any of: { "B15.9", "B16.0", "B16.9", "B17.10", "B17.2", "B17.9", "B18.8", "B19.11", "B19.20", "B19.9", "B25.1", "I85.01", "I85.10", "K70.10", "K70.11",

© 2023 Zhang Y et al. *JAMA Network Open*.

	“K70.40”, “K70.9”, “K71.0”, “K71.10”, “K71.11”, “K71.2”, “K71.3”, “K71.50”, “K71.6”, “K71.9”, “K72.00”, “K72.90”, “K72.91”, “K73.1”, “K73.2”, “K73.8”, “K74.0”, “K74.69”, “K75.1”, “K75.3”, “K75.89”, “K75.9”, “K76.0”, “K76.3”, “K76.4”, “K76.5”, “K76.9”, “K77”, “K83.5”, “R17”, “R18.8”, “B15.0”, “B16.1”, “B16.2”, “B17.0”, “B17.11”, “B17.8”, “B18.0”, “B18.1”, “B18.2”, “B18.9”, “B19.0”, “B19.10”, “B19.21”, “I85.00”, “I85.11”, “K70.0”, “K70.2”, “K70.30”, “K70.31”, “K70.41”, “K71.4”, “K71.51”, “K71.7”, “K71.8”, “K72.01”, “K72.10”, “K72.11”, “K73.0”, “K73.9”, “K74.1”, “K74.2”, “K74.3”, “K74.4”, “K74.5”, “K74.60”, “K75.0”, “K75.2”, “K75.4”, “K75.81”, “K76.1”, “K76.2”, “K76.6”, “K76.7”, “K76.81”, “K76.89”, “K83.8”, “K87”, “R18.0” }
**Stroke**	Diagnosis Code, ICD-9 is any of: { “433.0”, “433.01”, “433.1”, “433.10”, “433.11”, “433.2”, “433.21”, “433.80”, “434.0”, “434.9”, “434.90”, “434.91”, “436”, “430”, “431”, “433.00”, “433.20”, “433.3”, “433.30”, “433.31”, “433.8”, “433.81”, “433.9”, “433.90”, “433.91”, “434.00”, “434.01”, “434.1”, “434.10”, “434.11” }<br>Diagnosis Code, ICD-10 is any of: { “I60.00”, “I60.10”, “I60.12”, “I60.2”, “I60.31”, “I60.4”, “I60.7”, “I60.8”, “I60.9”, “I61.1”, “I61.3”, “I61.5”, “I61.8”, “I61.9”, “I63.00”, “I63.011”, “I63.012”, “I63.019”, “I63.09”, “I63.119”, “I63.12”, “I63.132”, “I63.19”, “I63.20”, “I63.212”, “I63.219”, “I63.231”, “I63.239”, “I63.311”, “I63.319”, “I63.321”, “I63.332”, “I63.342”, “I63.349”, “I63.39”, “I63.40”, “I63.412”, “I63.421”, “I63.422”, “I63.429”, “I63.441”, “I63.442”, “I63.49”, “I63.50”, “I63.511”, “I63.521”, “I63.522”, “I63.531”, “I63.532”, “I63.6”, “I63.8”, “I65.03”, “I65.09”, “I65.23”, “I65.9”, “I66.02”, “I66.11”, “I66.19”, “I66.22”, “I66.3”, “I60.01”, “I60.02”, “I60.11”, “I60.30”, “I60.32”, “I60.50”, “I60.51”, “I60.52”, “I60.6”, “I61.0”, “I61.2”, “I61.4”, “I61.6”, “I63.02”, “I63.031”, “I63.032”, “I63.039”, “I63.10”, “I63.111”, “I63.112”, “I63.131”, “I63.139”, “I63.211”, “I63.22”, “I63.232”, “I63.29”, “I63.30”, “I63.312”, “I63.322”, “I63.329”, “I63.331”, “I63.339”, “I63.341”, “I63.411”, “I63.419”, “I63.431”, “I63.432”, “I63.439”, “I63.449”, “I63.512”, “I63.519”, “I63.529”, “I63.539”, “I63.541”, “I63.542”, “I63.549”, “I63.59”, “I63.9”, “I65.01”, “I65.02”, “I65.1”, “I65.21”, “I65.22”, “I65.29”, “I65.8”, “I66.01”, “I66.03”, “I66.09”, “I66.12”, “I66.13”, “I66.21”, “I66.23”, “I66.29”, “I66.8”, “I66.9” }
**ESRD**	Diagnosis Code (Any Confinement Position), ICD-9 is any of: { “585.5”, “585.6” }<br>Diagnosis Code (Any Confinement Position), ICD-10 is any of: { “N18.5”, “N18.6” }
**GI Bleeding**	Diagnosis Code (Any Confinement Position), ICD-9 is any of: { “530.7”, “531.00”, “531.20”, “531.41”, “531.60”, “532.00”, “532.21”, “532.40”, “532.41”, “532.60”, “533.00”, “533.01”, “533.20”, “533.40”, “533.41”, “533.61”, “534.00”, “534.01”, “534.20”, “534.21”, “534.41”, “534.60”, “569.3”, “456.0”, “456.20”, “530.82”, “531.01”, “531.21”, “531.40”, “531.61”, “532.01”, “532.20”, “532.61”, “533.21”, “533.60”, “534.40”, “534.61”, “578.0”, “578.1”, “578.9” }<br>Diagnosis Code (Any Confinement Position), ICD-10 is any of: { “I85.01”, “K25.0”, “K25.4”, “K26.0”, “K26.2”, “K27.4”, “K27.6”, “K28.0”, “K28.2”, “K28.4”, “K28.6”, “I85.11”, “K25.2”, “K25.6”, “K26.4”, “K26.6”, “K27.0”, “K27.2”, “K62.5”, “K92.0”, “K92.1”, “K92.2” }
**Alcohol abuse or dependence**	Diagnosis Code ICD-9 is any of: { “291.2”, “291.81”, “291.9”, “303.0”, “303.01”, “303.90”, “305.0”, “571.1”, “571.2”, “291.0”, “291.1”, “291.3”, “291.4”, “291.5”, “291.8”, “291.82”, “291.89”, “303.00”, “303.02”, “303.03”, “303.9”, “303.91”, “303.92”, “303.93”, “305.00”, “305.01”, “305.02”, “305.03”, “357.5”, “425.5”, “571.0”, “571.3”, “E860.0”, “V11.3” }<br>Diagnosis Code, ICD-10 is any of: { "F10.121", "F10.129", "F10.14", "F10.151", "F10.180", "F10.21", "F10.220", "F10.221", "F10.231", "F10.250", "F10.259", "F10.26", "F10.27",

© 2023 Zhang Y et al. *JAMA Network Open*.

	"F10.921", "F10.929", "F10.950", "F10.959", "F10.96", "F10.97", "F10.980", "F10.981", "F10.982", "F10.988", "F10.99", "K70.10", "K70.11", "K70.40", "K70.9", "T51.0X1A", "T51.0X1D", "V11.3", "F10.10", "F10.120", "F10.150", "F10.159", "F10.181", "F10.182", "F10.188", "F10.19", "F10.20", "F10.229", "F10.230", "F10.232", "F10.239", "F10.24", "F10.251", "F10.280", "F10.281", "F10.282", "F10.288", "F10.29", "F10.920", "F10.94", "F10.951", "G62.1", "I42.6", "K70.0", "K70.2", "K70.30", "K70.31", "K70.41", "T51.0X1S" }
**Diabetes**	Diagnosis Code, ICD-9 is any of: { "249.11", "249.30", "249.31", "249.41", "249.61", "249.70", "249.81", "249.91", "250.03", "250.13", "250.20", "250.21", "250.32", "250.40", "250.42", "250.52", "250.60", "250.62", "250.71", "250.73", "250.80", "250.82", "250.90", "250.91", "250.93", "357.2", "362.01", "362.02", "362.03", "362.04", "366.41", "249.00", "249.01", "249.10", "249.20", "249.21", "249.40", "249.50", "249.51", "249.60", "249.71", "249.80", "249.90", "250.00", "250.01", "250.02", "250.10", "250.11", "250.12", "250.22", "250.23", "250.30", "250.31", "250.33", "250.41", "250.43", "250.50", "250.51", "250.53", "250.61", "250.63", "250.70", "250.72", "250.81", "250.83", "250.92", "362.05", "362.06" }<br>Diagnosis Code, ICD-10 is any of: { "E08.10", "E08.21", "E08.22", "E08.319", "E08.3211", "E08.3299", "E08.331", "E08.3311", "E08.3312", "E08.3319", "E08.339", "E08.3392", "E08.3412", "E08.3491", "E08.3493", "E08.3499", "E08.3519", "E08.3522", "E08.3523", "E08.3532", "E08.3539", "E08.3541", "E08.3542", "E08.3543", "E08.3549", "E08.3559", "E08.359", "E08.3591", "E08.3593", "E08.37X1", "E08.37X9", "E08.40", "E08.42", "E08.52", "E08.59", "E08.610", "E08.620", "E08.622", "E08.628", "E08.630", "E08.638", "E08.641", "E08.65", "E08.69", "E08.8", "E08.9", "E09.01", "E09.29", "E09.311", "E09.3211", "E09.329", "E09.3293", "E09.3299", "E09.331", "E09.3391", "E09.3393", "E09.341", "E09.3412", "E09.3419", "E09.3512", "E09.3522", "E09.3523", "E09.3532", "E09.3551", "E09.359", "E09.36", "E09.39", "E09.44", "E09.59", "E09.610", "E09.618", "E09.620", "E09.621", "E09.630", "E09.638", "E09.649", "E09.65", "E09.69", "E09.9", "E10.10", "E10.11", "E10.311", "E10.321", "E10.3211", "E10.3213", "E10.3219", "E10.3311", "E10.3313", "E10.339", "E10.341", "E10.3412", "E10.3419", "E10.349", "E10.3492", "E10.351", "E10.3512", "E10.3513", "E10.36", "E10.37X2", "E10.37X3", "E10.37X9", "E10.39", "E10.41", "E10.43", "E10.44", "E10.52", "E10.59", "E10.618", "E10.628", "E10.638", "E10.641", "E10.69", "E11.01", "E11.11", "E11.22", "E11.3211", "E11.3213", "E11.3219", "E11.329", "E11.3291", "E11.3292", "E11.331", "E11.3312", "E11.3319", "E11.3392", "E11.3393", "E11.3411", "E11.3413", "E11.3499", "E11.351", "E11.3511", "E11.3523", "E11.3529", "E11.3531", "E11.3549", "E11.3552", "E11.3559", "E11.3593", "E11.36", "E11.37X2", "E11.37X9", "E11.39", "E11.41", "E11.44", "E11.49", "E11.51", "E11.59", "E11.610", "E11.628", "E11.630", "E11.638", "E11.641", "E11.649", "E11.65", "E13.10", "E13.22", "E13.319", "E13.3211", "E13.3213", "E13.3219", "E13.3291", "E13.3299", "E13.331", "E13.3312", "E13.3313", "E13.3319", "E13.339", "E13.3393", "E13.3399", "E13.3413", "E13.3492", "E13.3493", "E13.351", "E13.3511", "E13.3512", "E13.3522", "E13.3539", "E13.3542", "E13.3543", "E13.3559", "E13.39", "E13.41", "E13.43", "E13.44", "E13.51", "E13.59", "E13.610", "E13.618", "E13.620", "E13.622", "E13.628", "E13.649", "E08.00", "E08.01", "E08.11", "E08.29", "E08.311", "E08.321", "E08.3212", "E08.3213", "E08.3219", "E08.329", "E08.3291", "E08.3292", "E08.3293", "E08.3313", "E08.3391", "E08.3393", "E08.3399", "E08.341", "E08.3411", "E08.3413", "E08.3419", "E08.349", "E08.3492", "E08.351", "E08.3511", "E08.3512", "E08.3513", "E08.3521", "E08.3529", "E08.3531", "E08.3533", "E08.3551",

© 2023 Zhang Y et al. *JAMA Network Open*.

	"E08.3552", "E08.3553", "E08.3592", "E08.3599", "E08.36", "E08.37X2", "E08.37X3", "E08.39", "E08.41", "E08.43", "E08.44", "E08.49", "E08.51", "E08.618", "E08.621", "E08.649", "E09.00", "E09.10", "E09.11", "E09.21", "E09.22", "E09.319", "E09.321", "E09.3212", "E09.3213", "E09.3219", "E09.3291", "E09.3292", "E09.3311", "E09.3312", "E09.3313", "E09.3319", "E09.339", "E09.3399", "E09.3413", "E09.349", "E09.3491", "E09.3493", "E09.3499", "E09.351", "E09.3511", "E09.3513", "E09.3519", "E09.3533", "E09.3542", "E09.3552", "E09.3553", "E09.3591", "E09.3592", "E09.3593", "E09.3599", "E09.37X1", "E09.37X9", "E09.40", "E09.41", "E09.42", "E09.43", "E09.49", "E09.51", "E09.52", "E09.622", "E09.628", "E09.641", "E09.8", "E10.21", "E10.22", "E10.29", "E10.319", "E10.3212", "E10.329", "E10.3291", "E10.3292", "E10.3293", "E10.3299", "E10.331", "E10.3312", "E10.3319", "E10.3391", "E10.3392", "E10.3393", "E10.3399", "E10.3411", "E10.3413", "E10.3491", "E10.3493", "E10.3499", "E10.3511", "E10.3519", "E10.359", "E10.37X1", "E10.40", "E10.42", "E10.49", "E10.51", "E10.610", "E10.620", "E10.621", "E10.622", "E10.630", "E10.649", "E10.65", "E10.8", "E10.9", "E11.00", "E11.10", "E11.21", "E11.29", "E11.311", "E11.319", "E11.321", "E11.3212", "E11.3293", "E11.3299", "E11.3311", "E11.3313", "E11.339", "E11.3391", "E11.3399", "E11.341", "E11.3412", "E11.3419", "E11.349", "E11.3491", "E11.3492", "E11.3493", "E11.3512", "E11.3513", "E11.3519", "E11.3521", "E11.3522", "E11.3532", "E11.3533", "E11.3539", "E11.3541", "E11.3542", "E11.3543", "E11.3551", "E11.3553", "E11.359", "E11.3591", "E11.3592", "E11.3599", "E11.37X1", "E11.37X3", "E11.40", "E11.42", "E11.43", "E11.52", "E11.618", "E11.620", "E11.621", "E11.622", "E11.69", "E11.8", "E11.9", "E13.00", "E13.01", "E13.11", "E13.21", "E13.29", "E13.311", "E13.321", "E13.3212", "E13.329", "E13.3292", "E13.3293", "E13.3311", "E13.3391", "E13.3392", "E13.341", "E13.3411", "E13.3412", "E13.3419", "E13.349", "E13.3491", "E13.3499", "E13.3513", "E13.3519", "E13.3521", "E13.3523", "E13.3529", "E13.3531", "E13.3532", "E13.3533", "E13.3541", "E13.3549", "E13.3551", "E13.3552", "E13.3553", "E13.359", "E13.36", "E13.40", "E13.42", "E13.49", "E13.52", "E13.621", "E13.630", "E13.638", "E13.641", "E13.65", "E13.69", "E13.8", "E13.9" }
**Health utilization**	
**ED visit**	CPT/HCPCS Procedure Code is any of: { "99281", "99285", "99288", "99282", "99283", "99284" }
**Hospitalization, days**	The occurrence of Any Inpatient Confinement

© 2023 Zhang Y et al. *JAMA Network Open*.

eTable 5. Sensitivity Analyses of Antipsychotic Medication Discontinuation Rate (Prescription Gap >7 Days) After Initiation for Infection-Related Hospitalization

	Crude discontinuation rate	IPW adjusted discontinuation rate	Fine&Gray adjusted discontinuation rate
**Time (days)**	**Haloperidol (%)**		
30	58.8 (55.3, 62.1)	59.5 (55.9, 62.8)	55.7 (55.6, 55.8)
60	85.5 (82.4, 88.1)	85.7 (82.6, 88.2)	77.0 (77.0, 77.1)
90	90.6 (87.7, 92.7)	90.6 (87.8, 92.8)	80.9 (80.9, 81.0)
180	96.4 (93.8, 97.9)	96.4 (93.9, 97.9)	85.4 (85.3, 85.4)
365	98.3 (95.9, 99.3)	98.3 (96.0, 99.3)	86.7 (86.7, 86.7)
**Time (days)**	**All atypical APMs (%)**		
30	14.5 (13.5, 15.5)	14.6 (13.6, 15.5)	14.2 (14.2, 14.2)
60	60.2 (58.6, 61.6)	60.3 (58.8, 61.8)	57.2 (57.1, 57.2)
90	70.2 (68.7, 71.6)	70.3 (68.8, 71.7)	66.3 (66.3, 66.4)
180	83.2 (81.8, 84.5)	83.3 (81.9, 84.5)	78.0 (78.0, 78.0)
365	90.8 (89.5, 91.9)	90.8 (89.5, 92.0)	84.7 (84.7, 84.7)

APM = antipsychotic medications, IPW=inverse probability weighting, Fine&Gray=Fine-Gray subdistribution hazard model

eTable 6. Sensitivity Analyses of Antipsychotic Medication Discontinuation Rate (Prescription Gap >30 Days) After Initiation for Infection-Related Hospitalization

	Crude discontinuation rate	IPW adjusted discontinuation rate	Fine&Gray adjusted discontinuation rate
**Time (days)**	**Haloperidol (%)**		
30	NA	NA	NA
60	70.7 (66.2, 74.6)	70.7 (66.2, 74.6)	65.6 (65.5, 65.7)
90	78.2 (73.9, 81.8)	78.3 (73.9, 81.9)	71.9 (71.9, 72.0)
180	90.8 (86.5, 93.7)	90.7 (86.4, 93.6)	81.5 (81.4, 81.6)
365	93.7 (88.7, 96.5)	93.9 (88.8, 96.6)	83.2 (83.1, 83.3)
**Time (days)**	**All atypical APMs (%)**		
30	NA	NA	NA
60	46.3 (44.7, 48.0)	46.3 (44.7, 47.9)	43.9 (43.9, 43.9)
90	54.6 (53.0, 56.3)	54.6 (52.9, 56.3)	51.6 (51.6, 51.6)
180	68.8 (67.1, 70.5)	68.8 (67.1, 70.5)	64.3 (64.3, 64.3)
365	77.6 (75.8, 79.2)	77.6 (75.8, 79.2)	71.7 (71.7, 71.7)

APM = antipsychotic medications, IPW=inverse probability weighting, Fine&Gray=Fine-Gray subdistribution hazard model

NA: discontinuation rate not applicable because sensitivity analysis with discontinuation defined as prescription gap > 30 days does not allow discontinuation at day 30 after CED

© 2023 Zhang Y et al. *JAMA Network Open*.

eTable 7. Sensitivity Analyses of Inverse Probability Weight–Adjusted Hazard Ratios of Antipsychotic Medication Discontinuation (Prescription Gap >7 Days) After Initiation for Infection-Related Hospitalization

	Haloperidol users	Atypical APM users
	HR* (95%CI)	HR* (95%CI)
**Age, years**		
**65-<75 *(Referent level)***	1.00	1.00
**75-<85**	0.91 (0.75, 1.11)	1.00 (0.92, 1.07)
**>=85**	1.12 (0.91, 1.36)	1.03 (0.94, 1.13)
**Gender**		
**Female *(Referent level)***	1.00	1.00
**Male**	0.94 (0.80, 1.09)	1.09 (1.02, 1.16)
**Race or ethnicity**		
**White *(Referent level)***	1.00	1.00
**Black**	1.01 (0.83, 1.24)	1.04 (0.95, 1.13)
**Others/missing**	1.12 (0.93, 1.36)	1.04 (0.96, 1.12)
**Frailty Score**		
**Robust *(Referent level)***	1.00	1.00
**Prefrail**	1.13 (0.70, 1.84)	0.99 (0.82, 1.20)
**Mildly frail**	0.96 (0.57, 1.61)	0.92 (0.76, 1.12)
**Moderate-to-severely frail**	0.90 (0.51, 1.60)	0.92 (0.74, 1.14)
**Infection type (reason for admission)**		
**COVID infection**	1.25 (0.82, 1.90)	1.05 (0.93, 1.19)
**Influenza**	0.62 (0.35, 1.10)	1.04 (0.79, 1.38)
**UTI**	1.01 (0.79, 1.29)	0.94 (0.85, 1.04)
**Pneumonia**	1.40 (1.10, 1.77)	1.07 (0.98, 1.18)
**Bacteremia**	0.92 (0.71, 1.21)	1.04 (0.92, 1.16)
**Endocarditis**	0.86 (0.13, 5.58)	1.01 (0.76, 1.34)
**Soft tissue infection**	1.19 (0.90, 1.57)	0.96 (0.85, 1.09)
**osteomyelitis/septic arthritis**	1.32 (0.89, 1.94)	1.16 (0.87, 1.54)
**CNS infections**	0.24 (0.02, 2.53)	0.93 (0.65, 1.33)
**Intra-abdominal infection**	1.35 (0.83, 2.19)	1.22 (0.95, 1.57)
**Comorbidities**		
**Dementia**	0.73 (0.61, 0.87)	0.82 (0.77, 0.88)
**Heart failure**	1.13 (0.95, 1.34)	1.02 (0.96, 1.10)
**Hypertension**	0.98 (0.83, 1.15)	1.04 (0.97, 1.11)
**Ischemic heart disease**	1.05 (0.86, 1.29)	0.87 (0.80, 0.95)
**Falls**	0.90 (0.74, 1.10)	1.03 (0.95, 1.11)
**CKD**	0.97 (0.82, 1.14)	1.04 (0.97, 1.12)
**Cancer**	1.04 (0.86, 1.27)	1.09 (1.00, 1.18)
**DVT**	1.19 (0.72, 1.98)	1.05 (0.88, 1.25)
**Anemia**	0.96 (0.81, 1.13)	0.99 (0.93, 1.06)

© 2023 Zhang Y et al. *JAMA Network Open*.

**Atrial fibrillation**	1.06 (0.90, 1.25)	1.00 (0.93, 1.07)
**Liver Disease**	0.99 (0.8, 1.23)	1.09 (0.99, 1.20)
**Stroke**	1.08 (0.90, 1.30)	0.91 (0.85, 0.98)
**ESRD**	1.12 (0.82, 1.53)	0.96 (0.83, 1.11)
**GI Bleeding**	1.30 (1.04, 1.63)	0.96 (0.86, 1.08)
**Alcohol abuse or dependence**	1.19 (0.89, 1.61)	0.91 (0.79, 1.04)
**Diabetes**	0.83 (0.71, 0.98)	1.04 (0.97, 1.11)
**Health utilization**		
**ED visit**	1.19 (0.96, 1.48)	1.02 (0.94, 1.10)
**Hospitalization, days**		
**<7 *(Referent level)***	1.00	1.00
**7-<30**	0.81 (0.69, 0.96)	0.97 (0.91, 1.04)
**>30**	0.64 (0.49, 0.83)	0.90 (0.81, 1.00)
**Cohort Entry Year**	1.05 (1.03, 1.07)	1.00 (0.99, 1.00)

*Adjusted for the covariates listed in this table using inverse probability weights

APM = antipsychotic medications, HR=Hazard Ratio, CI=confidence interval, COVID-19=coronavirus disease of 2019, UTI=urinary tract infection, CNS=central nervous system, CKD=chronic kidney disease, DVT=deep vein thrombosis, ESRD=end-stage renal disease, GI=gastrointestinal, ED visit=emergency department visit.

© 2023 Zhang Y et al. *JAMA Network Open*.

eTable 8. Sensitivity Analyses of Inverse Probability Weight–Adjusted Hazard Ratios of Antipsychotic Medication Discontinuation (Prescription Gap >30 Days) After Initiation for Infection-Related Hospitalization

	**Haloperidol users**	**Atypical APM users**
	HR* (95%CI)	HR* (95%CI)
**Age, years**		
**65-<75 *(Referent level)***	1.00	1.00
**75-<85**	1.00 (0.74, 1.34)	0.96 (0.88, 1.05)
**>=85**	1.09 (0.81, 1.46)	0.99 (0.89, 1.10)
**Gender**		
**Female *(Referent level)***	1.00	1.00
**Male**	0.80 (0.65, 0.97)	1.06 (0.98, 1.15)
**Race or ethnicity**		
**White *(Referent level)***	1.00	1.00
**Black**	1.10 (0.86, 1.41)	0.99 (0.88, 1.10)
**Others/missing**	1.00 (0.76, 1.33)	1.09 (0.99, 1.19)
**Frailty Score**		
**Robust *(Referent level)***	1.00	1.00
**Prefrail**	1.07 (0.53, 2.16)	1.08 (0.85, 1.38)
**Mildly frail**	0.90 (0.43, 1.87)	1.00 (0.77, 1.29)
**Moderate-to-severely frail**	0.79 (0.35, 1.76)	0.98 (0.74, 1.30)
**Infection type (reason for admission)**		
**COVID infection**	1.17 (0.63, 2.15)	1.15 (0.98, 1.34)
**Influenza**	1.09 (0.57, 2.10)	1.00 (0.70, 1.42)
**UTI**	1.15 (0.84, 1.58)	1.00 (0.89, 1.13)
**Pneumonia**	1.83 (1.33, 2.51)	1.15 (1.03, 1.29)
**Bacteremia**	0.89 (0.62, 1.26)	1.10 (0.95, 1.27)
**Endocarditis**	1.42 (0.78, 2.57)	1.07 (0.76, 1.52)
**Soft tissue infection**	1.37 (0.96, 1.96)	1.00 (0.86, 1.17)
**osteomyelitis/septic arthritis**	1.19 (0.59, 2.40)	1.38 (1.02, 1.86)
**CNS infections**	1.29 (0.61, 2.71)	1.23 (0.86, 1.76)
**Intra-abdominal infection**	1.49 (0.76, 2.96)	1.44 (1.11, 1.88)
**Comorbidities**		
**Dementia**	0.70 (0.56, 0.88)	0.78 (0.72, 0.85)
**Heart failure**	1.23 (0.98, 1.54)	0.99 (0.91, 1.08)
**Hypertension**	1.04 (0.84, 1.30)	1.00 (0.93, 1.09)
**Ischemic heart disease**	1.12 (0.88, 1.43)	0.91 (0.81, 1.01)
**Falls**	0.97 (0.75, 1.27)	0.99 (0.90, 1.09)
**CKD**	0.85 (0.68, 1.08)	1.06 (0.97, 1.16)
**Cancer**	1.08 (0.82, 1.41)	1.12 (1.02, 1.24)
**DVT**	0.90 (0.30, 2.71)	1.09 (0.86, 1.37)
**Anemia**	0.95 (0.76, 1.19)	1.03 (0.96, 1.12)

© 2023 Zhang Y et al. *JAMA Network Open*.

**Atrial fibrillation**	1.09 (0.88, 1.34)	1.06 (0.98, 1.15)
**Liver Disease**	1.01 (0.76, 1.36)	1.09 (0.97, 1.23)
**Stroke**	1.08 (0.85, 1.36)	0.92 (0.84, 1.00)
**ESRD**	1.35 (0.75, 2.45)	0.94 (0.77, 1.15)
**GI Bleeding**	1.40 (1.01, 1.94)	0.92 (0.80, 1.06)
**Alcohol abuse or dependence**	1.13 (0.73, 1.73)	0.93 (0.79, 1.09)
**Diabetes**	0.75 (0.60, 0.93)	0.99 (0.91, 1.07)
**Health utilization**		
**ED visit**	1.27 (0.97, 1.67)	0.95 (0.87, 1.04)
**Hospitalization, days**		
**<7 *(Referent level)***	1.00	1.00
**7-<30**	0.76 (0.62, 0.94)	0.94 (0.87, 1.02)
**>30**	0.61 (0.43, 0.89)	0.85 (0.74, 0.96)
**Cohort Entry Year**	1.05 (1.03, 1.07)	1.00 (0.99, 1.01)

*Adjusted for the covariates listed in this table using inverse probability weights

APM = antipsychotic medications, HR=Hazard Ratio, CI=confidence interval, COVID-19=coronavirus disease of 2019, UTI=urinary tract infection, CNS=central nervous system, CKD=chronic kidney disease, DVT=deep vein thrombosis, ESRD=end-stage renal disease, GI=gastrointestinal, ED visit=emergency department visit

© 2023 Zhang Y et al. *JAMA Network Open*.

eTable 9. Sensitivity Analyses of Antipsychotic Medication Discontinuation Rate After Initiation for Infection-Related Hospitalization, With 365 Days of Baseline Enrollment, Covariate Assessment Period, and Washout Period to Define New APM Use

	**Crude discontinuation rate***	**IPW adjusted discontinuation rate***
	**Haloperidol (%)**	
30	52.2 (48.1, 56.0)	52.9 (48.8, 56.7)
60	79.3 (75.4, 82.6)	79.5 (75.6, 82.8)
90	85.9 (82.2, 88.8)	86.0 (82.3, 88.9)
180	94.3 (90.8, 96.5)	94.5 (91.1, 96.6)
365	97.3 (93.2, 98.9)	97.5 (93.6, 99.0)
**Time (days)**	**All atypical APMs (%)**	
30	11.3 (10.3, 12.3)	11.4 (10.4, 12.3)
60	53.9 (52.2, 55.6)	54.1 (52.4, 55.7)
90	64.3 (62.6, 65.9)	64.5 (62.8, 66.1)
180	76.7 (75.0, 78.2)	76.8 (75.1, 78.3)
365	84.6 (82.9, 86.1)	84.6 (82.9, 86.1)

* Discontinuation was defined as prescription gap > 15

APM = antipsychotic medications, IPW=inverse probability weighting

© 2023 Zhang Y et al. *JAMA Network Open*.

eTable 10. Sensitivity Analyses of Hazard Ratios of Antipsychotic Medication Discontinuation After Initiation for Infection-Related Hospitalization, With 365 Days of Baseline Enrollment, Covariate Assessment Period, and Washout Period to Define New APM Use

	**Haloperidol users**	**Atypical APM users**
	HR* (95%CI)	HR* (95%CI)
**Age, years**		
**65-<75 *(Referent level)***	1.00	1.00
**75-<85**	1.02 (0.78, 1.33)	0.97 (0.89, 1.06)
**>=85**	1.21 (0.94, 1.58)	1.02 (0.92, 1.14)
**Gender**		
**Female *(Referent level)***	1.00	1.00
**Male**	0.81 (0.68, 0.96)	1.03 (0.95, 1.11)
**Race or ethnicity**		
**White *(Referent level)***	1.00	1.00
**Black**	1.03 (0.82, 1.30)	1.01 (0.91, 1.12)
**Others/missing**	1.10 (0.86, 1.40)	1.09 (1.00, 1.19)
**Frailty Score**		
**Robust *(Referent level)***	1.00	1.00
**Prefrail**	0.91 (0.52, 1.59)	1.03 (0.83, 1.29)
**Mildly frail**	0.79 (0.43, 1.42)	0.95 (0.76, 1.20)
**Moderate-to-severely frail**	0.68 (0.35, 1.33)	0.96 (0.75, 1.23)
**Infection type (reason for admission)**		
**COVID infection**	1.20 (0.75, 1.91)	1.08 (0.94, 1.26)
**Influenza**	0.83 (0.40, 1.72)	0.91 (0.64, 1.29)
**UTI**	0.88 (0.67, 1.16)	0.92 (0.82, 1.04)
**Pneumonia**	1.30 (0.99, 1.71)	1.09 (0.97, 1.21)
**Bacteremia**	1.02 (0.74, 1.39)	1.04 (0.91, 1.19)
**Endocarditis**	0.57 (0.12, 2.65)	1.16 (0.88, 1.54)
**Soft tissue infection**	1.14 (0.83, 1.55)	0.96 (0.83, 1.11)
**osteomyelitis/septic arthritis**	1.43 (0.82, 2.48)	1.27 (0.93, 1.72)
**CNS infections**	0.68 (0.30, 1.56)	1.08 (0.71, 1.63)
**Intra-abdominal infection**	1.35 (0.78, 2.34)	1.25 (0.95, 1.66)
**Comorbidities**		
**Dementia**	0.69 (0.56, 0.85)	0.80 (0.74, 0.87)
**Heart failure**	1.25 (1.02, 1.53)	1.04 (0.96, 1.12)
**Hypertension**	0.96 (0.79, 1.16)	1.02 (0.95, 1.11)
**Ischemic heart disease**	1.15 (0.89, 1.49)	0.93 (0.83, 1.04)
**Falls**	0.95 (0.76, 1.18)	0.98 (0.90, 1.07)
**CKD**	0.99 (0.81, 1.20)	1.09 (1.00, 1.18)
**Cancer**	1.20 (0.97, 1.48)	1.12 (1.02, 1.24)
**DVT**	1.25 (0.61, 2.57)	1.07 (0.89, 1.29)

© 2023 Zhang Y et al. *JAMA Network Open*.

**Anemia**	0.96 (0.79, 1.16)	1.01 (0.94, 1.09)
**Atrial fibrillation**	1.03 (0.85, 1.25)	1.04 (0.96, 1.13)
**Liver Disease**	1.03 (0.79, 1.34)	1.07 (0.96, 1.20)
**Stroke**	1.25 (1.02, 1.54)	0.91 (0.84, 0.99)
**ESRD**	1.03 (0.67, 1.57)	0.89 (0.74, 1.06)
**GI Bleeding**	1.25 (0.97, 1.61)	0.92 (0.81, 1.04)
**Alcohol abuse or dependence**	1.18 (0.78, 1.77)	0.92 (0.79, 1.08)
**Diabetes**	0.73 (0.60, 0.87)	1.03 (0.96, 1.11)
**Health utilization**		
**ED visit**	1.12 (0.85, 1.46)	0.99 (0.90, 1.09)
**Hospitalization, days**		
**<7 *(Referent level)***	1.00	1.00
**7-<30**	0.80 (0.65, 0.97)	0.93 (0.86, 1.00)
**>30**	0.57 (0.42, 0.77)	0.82 (0.73, 0.92)
**Cohort EntryYear**	1.05 (1.03, 1.07)	1.00 (0.99, 1.01)

*Adjusted for the covariates listed in this table using inverse probability weights. Discontinuation was defined as prescription gap > 15

APM = antipsychotic medications, HR=Hazard Ratio, CI=confidence interval, COVID-19=coronavirus disease of 2019, UTI=urinary tract infection, CNS=central nervous system, CKD=chronic kidney disease, DVT=deep vein thrombosis, ESRD=end-stage renal disease, GI=gastrointestinal, ED visit=emergency department visit.

© 2023 Zhang Y et al. *JAMA Network Open*.

eTable 11. Sensitivity Analyses of Antipsychotic Medication Discontinuation Rate After Initiation for Infection-Related Hospitalization, Without Censoring for Skilled Nursing Facility/Hospitalization During Follow-up

	**Crude discontinuation rate**	**IPW adjusted discontinuation rate**
	**Haloperidol (%)**	
30	49.9 (46.3, 53.2)	50.4 (46.8, 53.8)
60	78.6 (75.3, 81.5)	78.9 (75.6, 81.7)
90	85.8 (82.8, 88.2)	86.0 (83.1, 88.5)
180	93.9 (91.4, 95.6)	94.0 (91.6, 95.7)
365	97.4 (95.1, 98.6)	97.5 (95.2, 98.7)
**Time (days)**	**All atypical APMs (%)**	
30	11.1 (10.3, 11.9)	11.1 (10.3, 12.0)
60	55.3 (53.9, 56.6)	55.4 (54.0, 56.8)
90	66.5 (65.1, 67.8)	66.6 (65.2, 67.9)
180	79.2 (77.9, 80.4)	79.3 (78.0, 80.5)
365	87.8 (86.6, 88.8)	87.8 (86.7, 88.9)

* Discontinuation was defined as prescription gap > 15

APM = antipsychotic medications, IPW=inverse probability weighting

© 2023 Zhang Y et al. *JAMA Network Open*.

eTable 12. Sensitivity Analyses of Hazard Ratios of Antipsychotic Medication Discontinuation After Initiation for Infection-Related Hospitalization, Without Censoring for Skilled Nursing Facility/Hospitalization During Follow-up

	**Haloperidol users**	**Atypical APM users**
	Hazard Ratio*	Hazard Ratio*
**Age, years**		
**65-<75 *(Referent level)***	1.00	1.00
**75-<85**	1.10 (0.89, 1.35)	0.98 (0.91, 1.05)
**>=85**	1.27 (1.02, 1.57)	1.03 (0.95, 1.12)
**Gender**		
**Female *(Referent level)***	1.00	1.00
**Male**	0.89 (0.77, 1.04)	1.03 (0.97, 1.09)
**Race or ethnicity**		
**White *(Referent level)***	1.00	1.00
**Black**	1.19 (0.99, 1.44)	1.05 (0.97, 1.13)
**Others/missing**	1.03 (0.84, 1.27)	1.06 (0.98, 1.13)
**Frailty Score**		
**Robust *(Referent level)***	1.00	1.00
**Prefrail**	0.80 (0.52, 1.24)	0.90 (0.74, 1.10)
**Mildly frail**	0.71 (0.44, 1.13)	0.84 (0.68, 1.03)
**Moderate-to-severely frail**	0.72 (0.43, 1.23)	0.86 (0.69, 1.07)
**Infection type (reason for admission)**		
**COVID infection**	1.28 (0.83, 1.98)	1.05 (0.93, 1.19)
**Influenza**	0.54 (0.29, 0.98)	1.00 (0.75, 1.34)
**UTI**	0.99 (0.78, 1.26)	0.94 (0.86, 1.03)
**Pneumonia**	1.39 (1.10, 1.77)	1.08 (0.99, 1.18)
**Bacteremia**	0.97 (0.74, 1.28)	1.07 (0.96, 1.19)
**Endocarditis**	0.71 (0.18, 2.80)	1.00 (0.74, 1.34)
**Soft tissue infection**	1.25 (0.96, 1.62)	0.95 (0.85, 1.06)
**osteomyelitis/septic arthritis**	1.30 (0.85, 2.00)	1.24 (1.01, 1.53)
**CNS infections**	1.05 (0.54, 2.04)	0.97 (0.69, 1.35)
**Intra-abdominal infection**	1.39 (0.91, 2.14)	1.31 (1.06, 1.62)
**Comorbidities**		
**Dementia**	0.71 (0.59, 0.84)	0.81 (0.76, 0.86)
**Heart failure**	1.25 (1.05, 1.49)	1.02 (0.96, 1.09)
**Hypertension**	0.94 (0.80, 1.11)	1.05 (0.99, 1.12)
**Ischemic heart disease**	1.02 (0.82, 1.27)	0.93 (0.85, 1.01)
**Falls**	0.94 (0.77, 1.14)	1.04 (0.97, 1.11)
**CKD**	0.91 (0.77, 1.08)	1.05 (0.99, 1.13)
**Cancer**	1.06 (0.88, 1.29)	1.12 (1.03, 1.21)
**DVT**	0.90 (0.51, 1.59)	1.15 (1.00, 1.33)
**Anemia**	0.95 (0.81, 1.11)	0.98 (0.92, 1.04)

© 2023 Zhang Y et al. *JAMA Network Open*.

**Atrial fibrillation**	1.03 (0.87, 1.21)	1.02 (0.96, 1.09)
**Liver Disease**	1.02 (0.81, 1.27)	1.07 (0.98, 1.17)
**Stroke**	1.10 (0.92, 1.31)	0.92 (0.86, 0.98)
**ESRD**	1.03 (0.75, 1.43)	0.92 (0.80, 1.06)
**GI Bleeding**	1.30 (1.04, 1.62)	1.01 (0.92, 1.11)
**Alcohol abuse or dependence**	1.13 (0.81, 1.57)	0.88 (0.78, 1.01)
**Diabetes**	0.82 (0.70, 0.96)	1.05 (0.99, 1.11)
**Health utilization**		
**ED visit**	1.14 (0.91, 1.42)	1.01 (0.94, 1.08)
**Hospitalization, days**		
**<7 *(Referent level)***	1.00	1.00
**7-<30**	0.75 (0.64, 0.89)	0.98 (0.92, 1.05)
**>30**	0.66 (0.50, 0.86)	0.94 (0.85, 1.03)
**Cohort Entry Date, Year**	1.05 (1.03, 1.06)	1.00 (0.99, 1.00)

*Adjusted for the covariates listed in this table using inverse probability weights. Discontinuation was defined as prescription gap > 15

APM = antipsychotic medications, HR=Hazard Ratio, CI=confidence interval, COVID-19=coronavirus disease of 2019, UTI=urinary tract infection, CNS=central nervous system, CKD=chronic kidney disease, DVT=deep vein thrombosis, ESRD=end-stage renal disease, GI=gastrointestinal, ED visit=emergency department visit.

© 2023 Zhang Y et al. *JAMA Network Open*.

eReferences

1. Blatz AM, David MZ, Otto WR, Luan X, Gerber JS. Validation of International Classification of Disease-10 Code for Identifying Children Hospitalized With Coronavirus Disease-2019. *J Pediatric Infect Dis Soc*. Apr 30 2021;10(4):547-548. doi:10.1093/jpids/piaa140

2. Lin KJ, Dvorin E, Kesselheim AS. Prescribing systemic steroids for acute respiratory tract infections in United States outpatient settings: A nationwide population-based cohort study. *PLoS Med*. Mar 2020;17(3):e1003058. doi:10.1371/journal.pmed.1003058

3. Higgins TL, Deshpande A, Zilberberg MD, et al. Assessment of the Accuracy of Using ICD-9 Diagnosis Codes to Identify Pneumonia Etiology in Patients Hospitalized With Pneumonia. *JAMA Netw Open*. Jul 1 2020;3(7):e207750. doi:10.1001/jamanetworkopen.2020.7750

4. Star K, Bate A, Meyboom RH, Edwards IR. Pneumonia following antipsychotic prescriptions in electronic health records: a patient safety concern? *Br J Gen Pract*. Oct 2010;60(579):e385-94. doi:10.3399/bjgp10X532396

5. Ramgopal S, Noorbakhsh KA, Pruitt CM, Aronson PL, Alpern ER, Hickey RW. Outcomes of Young Infants with Hypothermia Evaluated in the Emergency Department. *J Pediatr*. Jun 2020;221:132-137 e2. doi:10.1016/j.jpeds.2020.03.002

6. Germanos G, Light P, Zoorob R, et al. Validating Use of Electronic Health Data to Identify Patients with Urinary Tract Infections in Outpatient Settings. *Antibiotics (Basel)*. Aug 25 2020;9(9)doi:10.3390/antibiotics9090536

7. Landers T, Apte M, Hyman S, Furuya Y, Glied S, Larson E. A comparison of methods to detect urinary tract infections using electronic data. *Jt Comm J Qual Patient Saf*. Sep 2010;36(9):411-7. doi:10.1016/s1553-7250(10)36060-0

8. Clemens JQ, Meenan RT, O'Keeffe Rosetti MC, Kimes T, Calhoun EA. Prevalence of and risk factors for prostatitis: population based assessment using physician assigned diagnoses. *J Urol*. Oct 2007;178(4 Pt 1):1333-7. doi:10.1016/j.juro.2007.05.140

9. Kim DH, Jeong SN, Lee JH. Chronic Periodontal Disease increases risk for Prostate Cancer in Elderly individuals in South Korea: a Retrospective Nationwide Population-based Cohort Study. *J Cancer*. 2020;11(16):4716-4723. doi:10.7150/jca.45369

10. Semins MJ, Shore AD, Makary MA, Weiner J, Matlaga BR. The impact of obesity on urinary tract infection risk. *Urology*. Feb 2012;79(2):266-9. doi:10.1016/j.urology.2011.09.040

© 2023 Zhang Y et al. *JAMA Network Open*.

11. Duarte Thibault M, Milen A, Burns L, Tilea A, Piehl K, Morgan D. Reduction in Rates of Symptomatic Urinary Tract Infection After Pelvic Reconstructive Surgery: A Quality Improvement Analysis. *Urogynecology (Hagerstown)*. Nov 1 2022;28(11):763-769. doi:10.1097/SPV.0000000000001242

12. Payne CK, Joyce GF, Wise M, Clemens JQ, Urologic Diseases in America P. Interstitial cystitis and painful bladder syndrome. *J Urol*. Jun 2007;177(6):2042-9. doi:10.1016/j.juro.2007.01.124

13. Kane BG, Degutis LC, Sayward HK, D'Onofrio G. Compliance with the Centers for Disease Control and Prevention recommendations for the diagnosis and treatment of sexually transmitted diseases. *Acad Emerg Med*. Apr 2004;11(4):371-7. doi:10.1197/j.aem.2003.11.016

14. Liu XQ, Wang CC, Liu YB, Liu K. Renal and perinephric abscesses in West China Hospital: 10-year retrospective-descriptive study. *World J Nephrol*. Jan 6 2016;5(1):108-14. doi:10.5527/wjn.v5.i1.108

15. Dotters-Katz SK, Heine RP, Grotegut CA. Medical and infectious complications associated with pyelonephritis among pregnant women at delivery. *Infect Dis Obstet Gynecol*. 2013;2013:124102. doi:10.1155/2013/124102

16. Qureshi AH, Ajayi O, Schwaderer AL, Hains DS. Decreased Identification of Vesicoureteral Reflux: A Cautionary Tale. *Front Pediatr*. 2017;5:175. doi:10.3389/fped.2017.00175

17. McGrew KM, Carabin H, Garwe T, et al. Validity of ICD-based algorithms to estimate the prevalence of injection drug use among infective endocarditis hospitalizations in the absence of a reference standard. *Drug Alcohol Depend*. Apr 1 2020;209:107906. doi:10.1016/j.drugalcdep.2020.107906

18. Shen HN, Lu CL. Skin and soft tissue infections in hospitalized and critically ill patients: a nationwide population-based study. *BMC Infect Dis*. Jun 4 2010;10:151. doi:10.1186/1471-2334-10-151

19. Lo Re V, 3rd, Carbonari DM, Jacob J, et al. Validity of ICD-10-CM diagnoses to identify hospitalizations for serious infections among patients treated with biologic therapies. *Pharmacoepidemiol Drug Saf*. Jul 2021;30(7):899-909. doi:10.1002/pds.5253

20. McCarthy NL, Baggs J, See I, et al. Bacterial Infections Associated With Substance Use Disorders, Large Cohort of United States Hospitals, 2012-2017. *Clin Infect Dis*. Oct 23 2020;71(7):e37-e44. doi:10.1093/cid/ciaa008

21. Massey PA, Feibel B, Thomson H, Watkins A, Chauvin B, Barton RS. Synovial fluid leukocyte cell count before versus after administration of antibiotics in patients with septic

arthritis of a native joint. *J Orthop Sci*. Sep 2020;25(5):907-910. doi:10.1016/j.jos.2019.11.011

22. Ho NT, Hoang VMT, Le NNT, et al. A spatial and temporal analysis of paediatric central nervous system infections from 2005 to 2015 in Ho Chi Minh City, Vietnam. *Epidemiol Infect*. Nov 2017;145(15):3307-3317. doi:10.1017/S095026881700228X

23. Gedeborg R, Furebring M, Michaelsson K. Diagnosis-dependent misclassification of infections using administrative data variably affected incidence and mortality estimates in ICU patients. *J Clin Epidemiol*. Feb 2007;60(2):155-62. doi:10.1016/j.jclinepi.2006.05.013

24. Zilberberg MD, Nathanson BH, Ditch K, Lawrence K, Olesky M, Shorr AF. Carbapenem Treatment and Outcomes Among Patients With Culture-Positive Complicated Intra-abdominal Infections in US Hospitals: A Retrospective Cohort Study. *Open Forum Infect Dis*. Dec 2019;6(12):ofz504. doi:10.1093/ofid/ofz504

25. Kim DH, Pawar A, Gagne JJ, et al. Frailty and Clinical Outcomes of Direct Oral Anticoagulants Versus Warfarin in Older Adults With Atrial Fibrillation : A Cohort Study. *Ann Intern Med*. Sep 2021;174(9):1214-1223. doi:10.7326/M20-7141

26. Kim DH, Schneeweiss S, Glynn RJ, Lipsitz LA, Rockwood K, Avorn J. Measuring Frailty in Medicare Data: Development and Validation of a Claims-Based Frailty Index. *J Gerontol A Biol Sci Med Sci*. Jun 14 2018;73(7):980-987. doi:10.1093/gerona/glx229

© 2023 Zhang Y et al. *JAMA Network Open*.
