# Supplementary material for: Antipsychotic Medication Use Among Older Adults Following Infection-Related Hospitalization
Source: JAMA Netw Open. 2023 Feb 17;6(2):e230063. doi: 10.1001/jamanetworkopen.2023.0063 (PMC9938426; doi:10.1001/jamanetworkopen.2023.0063)
Supplement: Supplement 2. — Data Sharing Statement [file jamanetwopen-e230063-s002.pdf]

# Data Sharing Statement

Zhang. Antipsychotic Medication Use Among Older Adults Following Infection-Related Hospitalization. *JAMA Netw Open*. Published February 17, 2023.
doi:10.1001/jamanetworkopen.2023.0063

**Data**
**Data available:** No

**Additional Information**
**Explanation for why data not available:** The data that support the findings of this study are available from a Commercial data vendor (Optum® Clinformatics®). Restrictions apply to the availability of these data, which were used under license for this study. Data are available from the author(s) with the permission of the Commercial data vendor. Although data cannot be shared with third parties, reasonable requests for reanalysis of the data will be considered by the authors.
